# Cholesterol homeostasis in the vertebrate retina: biology and pathobiology

**DOI:** 10.1194/jlr.TR120000979

**Published:** 2021-03-02

**Authors:** Sriganesh Ramachandra Rao, Steven J. Fliesler

**Affiliations:** 1 Departments of Ophthalmology and Biochemistry and Neuroscience Graduate Program, Jacobs School of Medicine and Biomedical Sciences, State University of New York- University at Buffalo, Buffalo, NY, USA; 2 Research Service, VA Western NY Healthcare System, Buffalo, NY, USA

**Keywords:** cholesterol, de novo synthesis, homeostasis, lipoprotein, oxysterol, photoreceptor, retina, retinal pigment epithelium, 7DHC, 7-dehydrocholesterol, A2E, N-Retinylidene-N-retinylethanolamine (2-[2,6-dimethyl-8-(2,6,6-trimethyl-1-cyclohexen-1- yl)-1*E*,3*E*, 5*E*,7*E*-octatetraenyl]-1-(2-hydroxyethyl)-4-[4-methyl-6-(2,6,6-trimethyl-1-cyclohexen-1-yl)-1*E*, 3E, 5*E*-hexatrienyl]-pyridinium), AMD, age-related macular degeneration, CD36, cluster of differentiation 36, CNV, choroidal neovascularization, DHCR7, 7-dehydrocholesterol reductase, DHCR24, 24-dehydrocholesterol reductase, ERG, electroretinogram, GCL, Ganglion cell layer, HMGCR, HMG-CoA reductase, iPSC, induced pluripotent stem cell, IS, inner segment, LAL, lysosomal acid lipase, LOX-1, OxLDL receptor, LRP1, LDL-related particle 1, MVK, mevalonate kinase, NPC-I/II, Niemann-Pick type C I and II, OS, outer segment(s), PFO, perfringolysin O, PN, postnatal, ROS, reactive oxgen species, RPE, retinal pigment epithelium, SQLE, squalene monooxygenase, SRB-I/II, class B scavenger receptor (I and II), TSPO, translocator protein 18 kDa, VEGF, vascular endothelial growth factor

## Abstract

Cholesterol is a quantitatively and biologically significant constituent of all mammalian cell membrane, including those that comprise the retina. Retinal cholesterol homeostasis entails the interplay between de novo synthesis, uptake, intraretinal sterol transport, metabolism, and efflux. Defects in these complex processes are associated with several congenital and age-related disorders of the visual system. Herein, we provide an overview of the following topics: (a) cholesterol synthesis in the neural retina; (b) lipoprotein uptake and intraretinal sterol transport in the neural retina and the retinal pigment epithelium (RPE); (c) cholesterol efflux from the neural retina and the RPE; and (d) biology and pathobiology of defects in sterol synthesis and sterol oxidation in the neural retina and the RPE. We focus, in particular, on studies involving animal models of monogenic disorders pertinent to the above topics, as well as in vitro models using biochemical, metabolic, and *omic* approaches. We also identify current knowledge gaps and opportunities in the field that beg further research in this topic area.

Sterols represent a diverse class of biologically significant lipids that are found ubiquitously in all eukaryotic cells, primarily in the plasma membrane (1). Cholesterol is, by far, the dominant sterol normally found in mammalian cells and tissues. Maintaining optimal levels of cholesterol is a requisite for normal cellular function and viability, and represents a delicate balance between endogenous de novo synthesis, exogenous uptake, and efflux of sterols. Mechanisms governing the process of cholesterol homeostasis have been investigated extensively, given the role they play both in normal biology and in several significant human clinical disorders, such as Alzheimer's disease, cardiovascular disease, and age-related macular degeneration (AMD) (2). Herein, we review the mechanisms governing cholesterol homeostasis in the neural retina, and the pathological mechanisms that underlie certain ocular diseases where this homeostasis is disturbed.

Cholesterol in the central nervous system (of which the retina is a part) exists mostly in the unesterified form, in nerve myelin and in the plasma membranes of both neuronal and non-neuronal (e.g., glial) cells. The brain relies exclusively on its own de novo synthesis of sterols because the blood-brain barrier excludes circulating lipoproteins (3). By comparison, sterol homeostasis in the retina is somewhat more complex because its sterol pool is derived from both local de novo synthesis and extraretinal uptake. The retina has many advantages for experimental studies, such as its layered organization and ease of accessibility, and represents one of the best-studied parts of the central nervous system. Cholesterol and its biogenic sterol precursors can undergo both enzymatic and nonenzymatic oxidation, which is particularly relevant, given the pro-oxidative environment of the retina, yielding a variety of oxysterol products, some of which are highly toxic to cells (4, 5). Sterols and sterol metabolites may play causative roles in several neurodegenerative conditions, including certain retinopathies (5, 6, 7).

In part, this review is an extension of a prior review in a similar Thematic Issue series in this journal published a decade ago (8). A subsequent review by other authors highlighted the role of sterol homeostatic processes in AMD (9). The scope of this review encompasses the following topics: (a) cholesterol biosynthesis in the neural retina (in vitro and in vivo isotopomer techniques, pharmacological inhibition of sterol biosynthesis, and pharmacological/genetic modeling of monogenic diseases affecting cholesterol biosynthesis); (b) cholesterol up-take by the neural retina and the retinal pigment epithelium (RPE), and intraretinal sterol transport (in vitro and in vivo modeling of lipoprotein uptake and monogenic disease affecting lipoprotein synthesis/uptake, LDL-tagging methods); (c) cholesterol efflux in the neural retina (role of LXRα/β, ATP binding cassette (ABC) transporters, cytochrome P450 (CYP) enzymes, related monogenic diseases, and mechanisms of drusen formation in AMD, isotopic and pharmacologic approaches to measure the retinal sterol turnover rate); and (d) biology of lipid peroxidation in the neural retina and the RPE, and the effects of oxysterols and oxidized LDL (OxLDL) in vitro and in vivo (uptake and metabolism of OxLDL and oxysterols and its relevance in retinopathies).

We will focus on studies that, for the most part, have been conducted using either laboratory animals (e.g., mice, rats) or animal-derived cells in vitro, augmented by the use of selective inhibitors of enzymes in the cholesterol synthesis pathway or by genetic manipulation of the genes encoding those enzymes. Although these experimental systems are admittedly highly simplified compared with the complexity of human ocular anatomy and physiology and cannot fully model multifactorial disorders such as AMD, they nonetheless have provided fundamental insights into cholesterol homeostasis in the vertebrate retina. In addition, the knowledge derived from such models has provided potentially useful tools for developing effective therapeutic interventions for human diseases that are caused by defects in the cholesterol pathway that impact the structure and function of the retina.

## Overview of retinal architecture

**Fig. 1:**
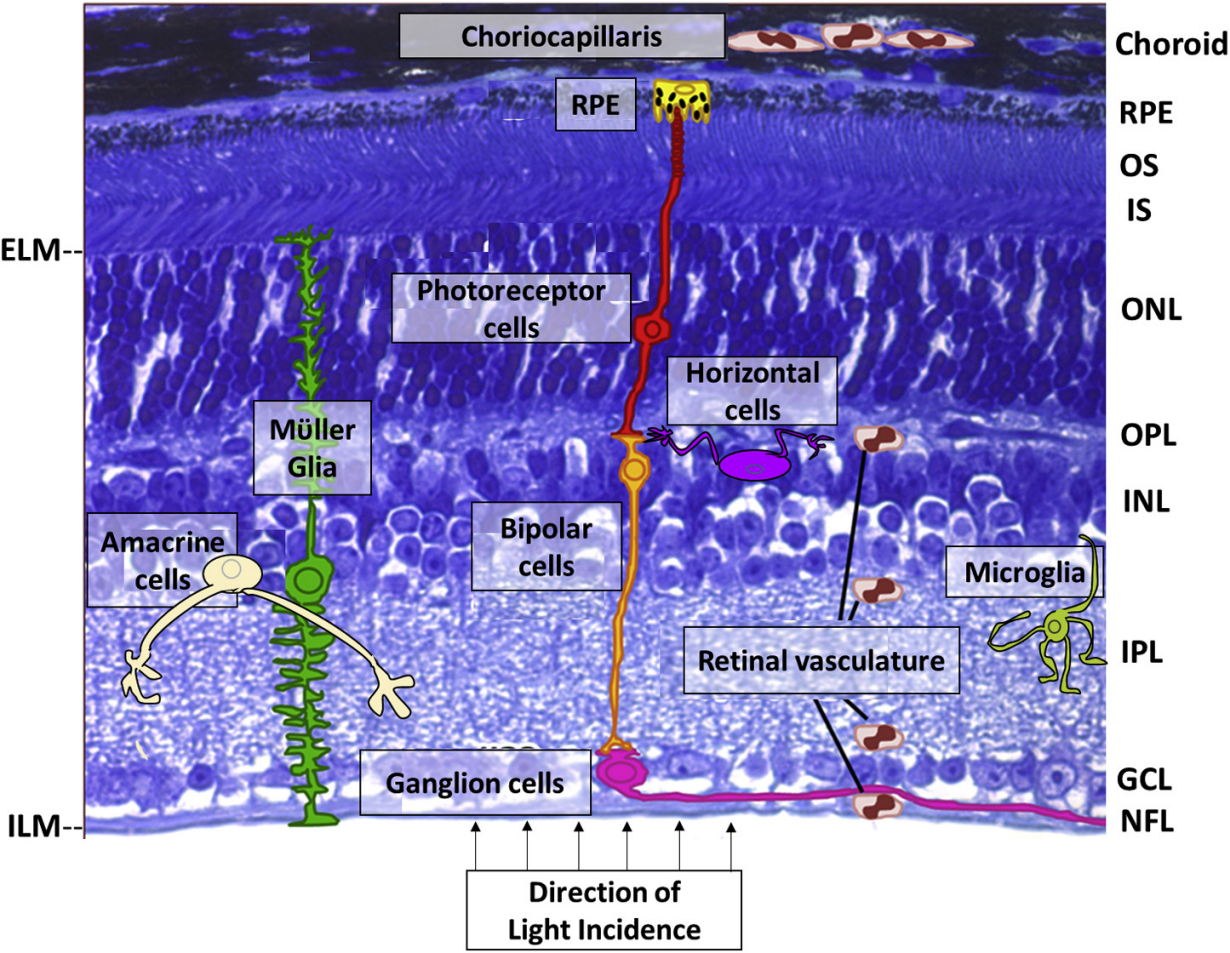
Histological organization of a model vertebrate retina. Schematic representation of individual retinal cell types is superimposed on a light microscopy image of a normal C57Bl/6J mouse retina (Toluidine blue-stained). The retinal pigment epithelium (RPE) forms a cellular monolayer interface between the neural retina and the choriocapillaris (the elements of the choroidal blood supply most proximal to the RPE). The RPE junctional complex network comprises the outer blood-retinal barrier, restricting the flow of blood-borne substances from the choroid to the outer retina. The photoreceptor layer (containing rods and cones) spans nearly half the total neural retina thickness and is comprised of the photoreceptor outer segment (OS) and inner segment (IS) layers, the outer nuclear layer (ONL, containing the rod and cone nuclei), and the outer plexiform layer (OPL), the latter containing the axonal processes and presynaptic endings of the photoreceptor cells, along with the postsynaptic processes of the bipolar cells and dendritic extensions of the horizontal cells. The inner nuclear layer (INL) consists of the nuclei and cell bodies of bipolar cells, amacrine cells, and horizontal cells, as well the Müller glia. The inner plexiform layer (IPL) consists of the axonal processes and synaptic termini of bipolar and amacrine cells, along with the dendritic arbors of the ganglion cells; the latter form the ganglion cell layer (GCL) of the neural retina. The collective axons of the ganglion cells form the nerve fiber layer (NFL) and exit the eye as the optic nerve en route to the visual cortex of the brain. The inner retinal cells are nourished by the retinal vasculature; the tight junctions of its constituent endothelial cells comprise the inner blood-retinal barrier. Microglia normally reside in the IPL and GCL, but migrate into the INL and outer retinal layers when activated. Note: The schematic does not depict some features specific to human or primate retinas, such as the macula or cone-rich fovea. ELM, external limiting membrane; ILM, internal limiting membrane. (Modified and adapted, with permission, from (10)).

The neural retina is the photon-sensory tissue lining the inner posterior segment of the eye (see Fig. 1, and http://webvision.med.utah.edu/). There are about 150 million cells in a human retina, about 85% of which are neurons, including, c.a., six to seven million cone photoreceptors and, c.a., 110–125 million rod photoreceptors. Photoreceptor cells are anatomically segregated into “inner segment” (IS) and “outer segment” (OS) compartments; the IS consists of mitochondria and houses the biosynthetic machinery of the cell, whereas the OS serves as the membrane residence for the phototransduction cascade (11). Other neuronal cell types in the retina include the bipolar, horizontal, amacrine, and ganglion cells, forming the neuronal circuitry involved in ultimately relaying visual information originating in the photoreceptor cells to the brain. The nonneuronal cell types include Müller glia, microglia, astrocytes, and the RPE, involved in meeting tissue homeostatic requirements. This review discusses the roles played by these cell types in retinal sterol homeostasis.

The OS membrane is comprised of approximately equal amounts of lipids and proteins, by weight (12): the dominant lipids are glycerophospholipids (80%–85%), whereas cholesterol represents only about 8–10 mol % of the total lipid (which is only about a third of the level of cholesterol found in the plasma membrane of most cells); the overwhelming majority of the protein content of OS membranes (>90%) is accounted for by the visual pigment apoprotein, opsin. Diurnally shed photoreceptor outer segment disk membranes are then phagocytized and degraded by the adjacent underlying RPE (13, 14). The compensatory synthesis and incorporation of OS membranes at its base contributes to the large demand for membrane constituents (including sterols) in photoreceptors (14). Such demands of the retina may be met by a combination of de novo synthesis and receptor-mediated uptake from two separate blood supplies—the choroidal vasculature (supplying the outer retina, notably the photoreceptor cells) and the inner retinal vasculature—in conjunction with an internal auxiliary source represented by Müller glia. In addition, synaptic connections in the outer and inner plexiform layers, respectively, also necessitate a high rate of turnover of cholesterol pools in retinal neurons because of assembly and recycling of the synaptic vesicles that contain neurotransmitters. Below, we will consider the various potential contributors to overall cholesterol homeostasis in the retina.

## General considerations: cholesterol synthesis, uptake, and efflux

**Fig. 2:**
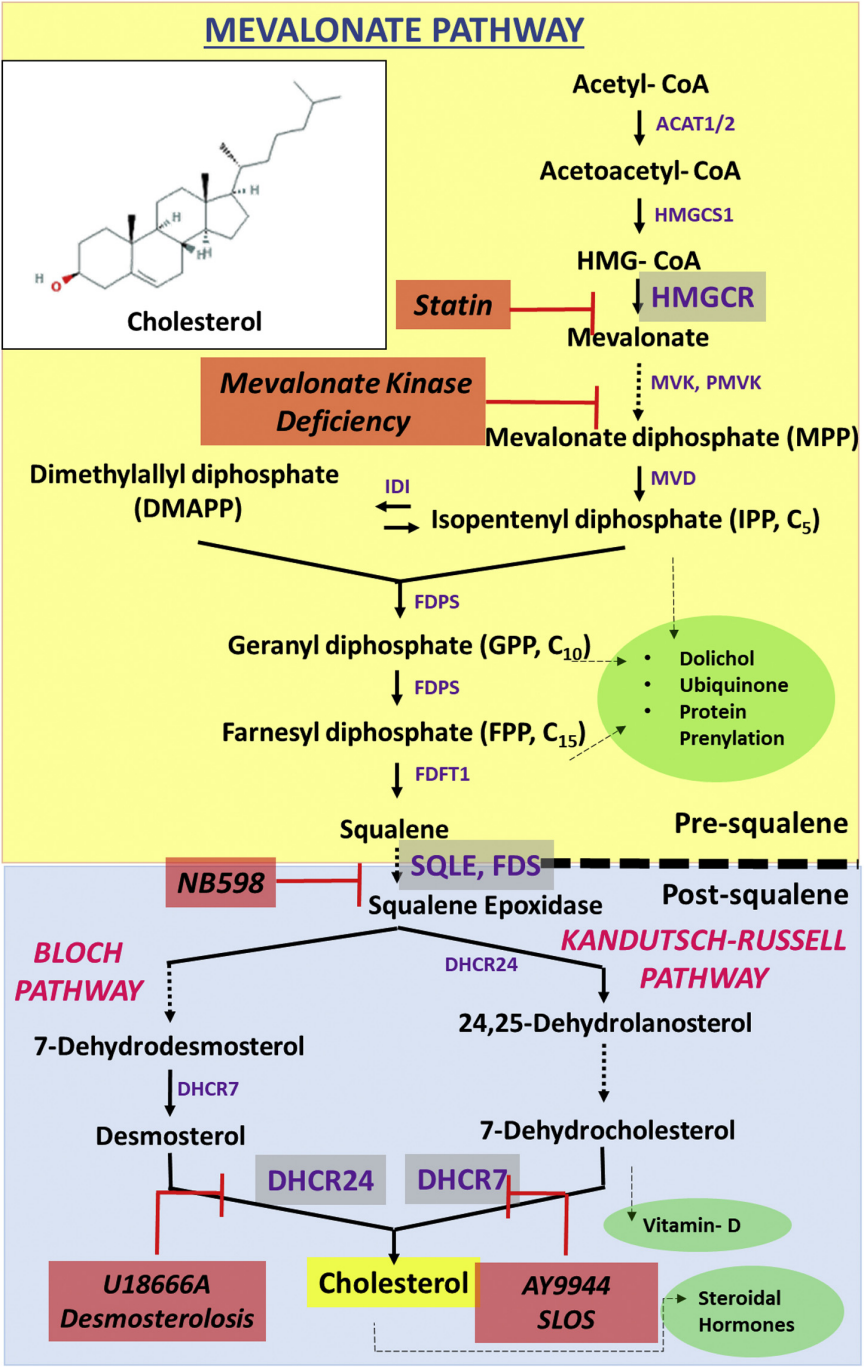
Schematic representation of the mevalonate pathway. Acetyl-CoA is converted in two steps, sequentially catalyzed by ACAT1 and ACAT2 and HMGCS1 (HMG-CoA synthase 1), to mevalonate, whose formation is the main rate-limiting step in the pathway, catalyzed by HMGCR (HMG-CoA reductase; inhibited by statins). The presqualene portion of the pathway generates a series of acyclic isoprenoid compounds, with a critical branch point at the level of farnesyl diphosphate (FPP) generation. The committed step toward sterol synthesis involves epoxidation of squalene to squalene-2,3-epoxide, catalyzed squalene epoxidase (SQLE; inhibited by NB-598), which then undergoes cyclization to form the first sterol intermediate (lanosterol; 4α,4β,14α-trimethyl-cholesta-8(9),24-dien-3β-ol) in the postsqualene portion of the pathway. This is followed by a series of demethylation and double-bond isomerization and reduction reactions, with ultimate engagement of either the Bloch Pathway or the Kandutsch-Russell Pathway to form cholesterol. Reduction of the side-chain double bonds in desmosterol (cholesta-5,24-dien-3β-ol) and 7-dehydrodesmosterol (cholesta-5,7,24-trien-3β-ol) is catalyzed by DHCR24 (inhibited by U18666A), whereas reduction of the ring B nuclear double bond in 7-dehydrocholesterol (7DHC; cholesta-5,7-dien-3β-ol) and 7-dehydrodesmosterol are catalyzed by DHCR7 (inhibited by AY9944). The mevalonate pathway is involved in the synthesis of several other important isoprenoid metabolites, including ubiquinone (coenzyme Q), dolichols, vitamin-D, and steroid hormones. Mutations in the DHCR24 gene lead to desmosterolosis, whereas such defects in the DHCR7 gene cause Smith-Lemli-Opitz syndrome (SLOS). Inset: Chemical structure of cholesterol (cholest-5-en-3β–ol). DHCR24, 24-dehydrocholesterol reductase; DHCR7, 7DHC reductase.

A general schematic of the cholesterol biosynthesis pathway is provided in Fig. 2. The rate-limiting step of the cholesterol synthesis pathway (a.k.a. the mevalonate pathway) is catalyzed by HMG-CoA reductase (HMGCR; OMIM# 142910, EC 1.1.1.88). A secondary regulatory locus in this pathway is at the level of squalene-2,3-epoxidase [a.k.a. squalene monooxygenase (SQLE) OMIM# 602019, E.C. 1.14.99.7] (15).

The mevalonate pathway generates linear isoprenoid products such as farnesyl diphosphate and geranylgeranyl diphosphate (used for prenylation of proteins), dolichol and its phosphorylated derivatives (required for protein *N*-glycosylation), and squalene (the committed acyclic intermediate required for sterol synthesis), to name a few (16, 17, 18). Sterol intermediates are then generated by cyclization of squalene through the Kandutsch-Russell pathway [lathosterol, 7-dehydrocholesterol (7DHC)] and the Bloch pathway (lanosterol, 7-dehydrodesmosterol, desmosterol). Desmosterol and 7DHC undergo reduction of double bonds at the C24 (Δ^24^) and C7 (Δ^7^) positions by 7DHC reductase (DHCR7; OMIM# 602858, EC 1.3.1.21) and 24-dehydrocholesterol reductase (DHCR24; OMIM# 606418, EC 1.3.1.72), respectively, to generate cholesterol. Genetic mutations in any step of this pathway can result in pathologies, typically recessive and often lethal, because of the buildup of intermediate sterols and their metabolites (19, 20). Examples of such congenital disorders and the affected enzymes include Smith-Lemli-Opitz syndrome (SLOS; DHCR7, OMIM# 270400), desmosterolosis (DHCR24, OMIM# 602398), lathosterolosis (sterol-C5-desaturase, OMIM# 607330), and mevalonate kinase (MVK, OMIM# 251170) deficiency (see Fig. 2) (19). The impact of such defects on the structure and function of the retina will be discussed.

As an alternative to de novo synthesis of cholesterol, cells may import cholesteryl esters by membrane receptor–mediated endocytosis of blood-borne, liver-derived lipoproteins (VLDL, LDL, IDL) (21, 22). Lysosomal acid lipase (LAL) generates free cholesterol in the lysosomal lumen, which is then trafficked to the ER by Niemann-Pick type C I and II (NPC-I/NPC-II) protein complex. Mutations in NPC-I/NPC-II, causing Niemann-Pick disease, alter cholesterol trafficking and subsequent accumulation of free cholesterol in lysosomes (20, 23). Alternatively, cholesterol uptake can involve class B scavenger receptor I (SRB-I)–mediated selective uptake of cholesteryl esters from HDL particles. OxLDL is endocytosed by the cell via cluster of differentiation 36 (CD36) and OxLDL receptor (LOX-1) receptors. The role of these sterol uptake machineries in the neural retina and the RPE is discussed.

Cellular cholesterol is effluxed to naïve apolipoprotein (APO)A1-containing HDL by ABC transporters, which represent a large class of biologically important molecules involved in efflux of diverse substrates, such as ions, peptides and proteins, membrane lipids, and lipid-soluble molecules (24, 25). Several of these, including ABCA1, ABCG1, and ABCG4, play important roles in cholesterol efflux from peripheral tissues to naïve APO-A1–containing HDL particles and, to a lesser extent, to APO-E–containing LDL particles (25, 26, 27). Defects in ABC transporters can result in severe, often chronic, pathologies [including those that impact the structure and function of the retina (see below)]; for example, defects in ABCA1-mediated efflux have been implicated in atherogenesis and neurological disorders, such as Alzheimer's disease (25, 28, 29). Tangier disease (OMIM# 205400) is caused by recessive mutations in ABCA1 and is characterized by hypoalphalipoproteinemia (low serum APO-A1-HDL levels), mild neuropathy, atherosclerosis, retinopathy, corneal infiltrates and scarring, lipid deposits in the conjunctiva, and cataract formation (30). (The reader is referred to a recent review that provides detailed analysis of the specific role of HDL in age-related retinopathies (31).) This review discusses the role of sterol efflux in retinal physiology and pathophysiology.

The transcriptional regulation of sterol efflux-related ABC transporters occurs through the LXR-α response element (32). Endogenous LXR-α agonists and antagonists play an important modulatory role in cellular sterol efflux (33, 34). Sterols undergo CYP enzyme-mediated (CYP27A1, CYP46A1, CYP11A1, CYP39A1, CYP7A1) oxidation or hydroxylation, generating sterol metabolites, which function as potent LXR-α agonists (35, 36). LXR activation stimulates sterol efflux by upregulating the expression of ABC transporters (37, 38). Mutations in a critical mitochondrial CYP enzyme, CYP27A1, causes cerebrotendinous xanthomatosis (OMIM# 213700), an autosomal recessive disorder clinically characterized by chronic diarrhea, neuronal dysfunction, early-onset of atherosclerosis, and visual system deficits such as cataract formation (39, 40). The CYP enzymes act on a broad spectrum of substrates including sterols and oxysterols, generating biologically important metabolites. CYP7A1 is known to metabolize 7DHC to 7-oxocholesterol and 7-ketocholesterol (7KChol), contributing to SLOS and cerebrotendinous xanthomatosis pathology (41, 42). Mitochondrial CYP27A1 metabolizes cholesterol to 27-COOH-Chol and 27-OH-Chol, and 7KChol to 27-COOH-7KChol and 27-OH-7KChol, via acid hydroxylation (38, 43), which stimulate ABCA1/G1-mediated cellular cholesterol efflux as discussed below (44, 45). Hereby, we discuss the dynamic interplay of de novo synthesis, uptake, efflux, and metabolism of cholesterol in the retina to maintain steady-state content of cholesterol (i.e., cholesterol homeostasis), and retinopathies that arise upon perturbing these important homeostatic processes.

## Retinal de novo cholesterol synthesis and uptake

### Insights into cholesterol synthesis and uptake in the neural retina

**Fig. 3:**
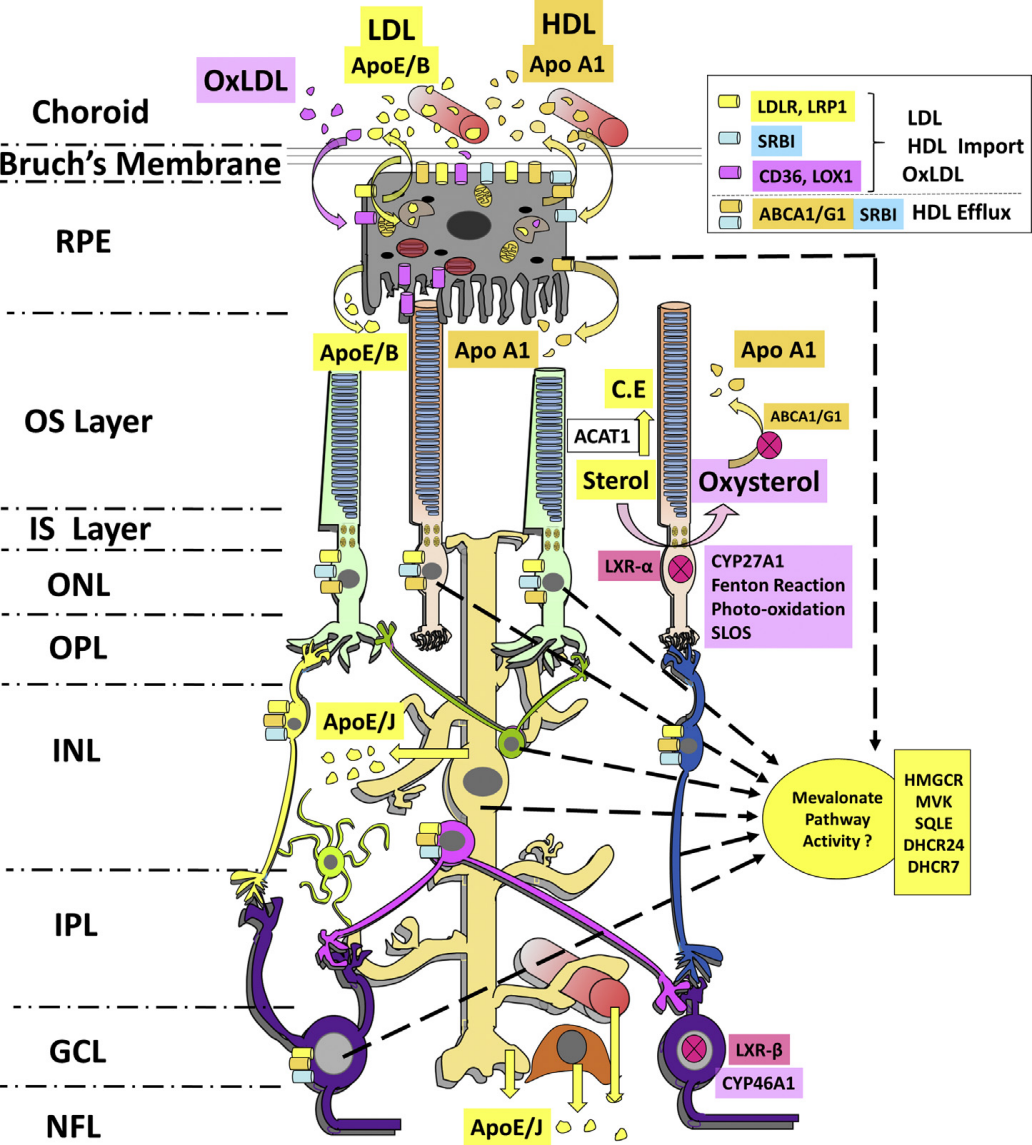
Hypothetical model of cholesterol homeostatic processes governing the vertebrate retina. The mevalonate pathway is active in both the RPE and the neural retina; however, the exact contributions of each of the retinal cell types to the overall synthesis and steady-state content of cholesterol in the retina remains to be determined. The RPE is capable of ABCA1-mediated bidirectional sterol efflux. The RPE also may exhibit apical secretion of APO-E–containing LDL, as well as LDLR-dependent uptake of LDL, and CD36-dependent uptake of OxLDL from the choroid. CD36 is also involved in diurnal uptake of rod outer segment (OS) tips; however, lipid hydroperoxides and oxysterols may competitively inhibit this process. Müller glia actively synthesize, package (with APO-E and APO-J), and secrete cholesterol, which then can be taken up by neighboring neurons. Sterol efflux from the neural retina is dependent on the activities of CYP27A1, CYP46A1, LXRs, and ABCA1. Excess retinal cholesterol may be esterified and stored as lipid droplets by the activity of ACAT1 and LCAT. Oxidative stress, involving both enzymatic and nonenzymatic processes, can lead to oxysterol formation; those by-products either are removed from the cell by sterol efflux or remain and accumulate in lipid droplets and cellular membranes, which can result in retinal pathology. (See Fig. 2 and text for definition of abbreviations.)

Early in vitro experiments by Fliesler and Schroepfer, using either bovine retina cell-free homogenates (10,000*g* supernatant fraction that contains microsome and cytosol) (46) or intact whole bovine retinas in organ culture (47) incubated with [^3^H]mevalonate, demonstrated sterol synthesis in the vertebrate neural retina. However, the radiolabel was primarily incorporated in mevalonate pathway intermediates such as 15- and 20-carbon isoprenoid acids, while conversion to cholesterol was rather limited. The first in vivo investigations of cholesterol synthesis in the vertebrate retina utilized intravitreal injection of [^3^H]acetate in rats followed by monitoring its incorporation into cholesterol, in the presence and absence of lovastatin, an inhibitor of HMGCR (48, 49). The neural retina showed [^3^H]cholesterol formation within 6 h, with little accumulation in intermediates, and its formation was fully inhibited upon coinjection with lovastatin (48, 49, 50). In the same study, inhibiting the postsqualene phase of the pathway using NB-598 (an inhibitor of squalene 2–3 epoxidase) (SQLE; Fig. 3) caused the accumulation of radiolabeled squalene, as predicted. Similarly, [^3^H]farnesol injected intravitreally in rats resulted in formation of [^3^H]cholesterol, in an NB-598–sensitive manner (51). These results first qualitatively demonstrated the presence of a functional de novo sterol synthesis pathway in the whole retina. However, calculation of absolute rates of cholesterol synthesis using this metabolic approach is not possible because of nonuniform cellular uptake and incorporation of radiolabeled de novo precursors into cholesterol, acetyl-CoA hydrolysis, and the pleiotropic effects of statins (52, 53, 54). Intravitreal injection of lovastatin led to early changes in the structural organization of the neural retina characterized by formation of rosette-like arrangements of photoreceptors and eventually necrosis of the retina by 4 days (49, 50). However, contrary to initial expectations, such effects of lovastatin, were found to be due to defective protein prenylation in the retina, rather than to disruption of cholesterol synthesis (55). Such pharmacological targeting of HMGCR and SQLE also provided critical evidence for a functional presqualene and postsqualene pathways in the rodent retina. To date, there have been no published studies regarding the operation of the mevalonate shunt pathway in the retina.

*In vivo* measurements of absolute rates of tissue cholesterol synthesis are achieved by chronic administration of deuterated water ([^2^H]water) and subsequent MS analysis of the cholesterol isotopomer distribution (56, 57, 58, 59). A recent investigation adapting the isotopomer approach suggested that majority (>70%) of retinal sterol arises from de novo synthesis (60). However, proper estimation of tissue sterol synthesis rates using this technique requires detailed assessment of several critical factors, such as molar fraction distribution in the tissue, molar enrichment in deuterated sterol (average number of [^2^H] atoms per newly synthesized sterol molecule), experimental verification of steady-state synthesis of [^2^H]cholesterol, and experimental determination of the correction factor to account for any newly synthesized cholesterol without [^2^H] incorporation (56, 57, 58, 59). Parallel quantification of retinal cholesterol uptake was measured in mice maintained on chow supplemented with 0.3% w/w [^2^H]cholesterol for 2 weeks. Sterol uptake in the retina (after 1 week) was estimated to be about 3.6% of the total cholesterol content (60). This experimental approach would be significantly strengthened by inclusion of a weaning experiment (i.e., weaning from [^2^H]water after 2 weeks back to normal water [t = 0]) to experimentally determine the true half-life (and hence, the absolute turnover rate) of labeled cholesterol in the retina.

Systemically administered simvastatin was shown to exhibit the highest bioavailability compared with other statins (after 6 weeks) in the neural retina of mice (61) and also was significantly higher than that of the brain tissue, suggesting that simvastatin is permeable to the blood-retinal barrier. Such treatment of adult mice led to a significant decrease (by about 20%, after 6 weeks) in retinal cholesterol content, as well as a reduction in sterol intermediates, but did not alter total retinal cholesterol uptake. Given the estimated cholesterol turnover rate (c.a., 54 days) in the retina, and the estimated contribution of endogenous (retina-derived) biosynthesis to the total retinal cholesterol pool (c.a., 72%) (60), it was concluded that systemic simvastatin treatment led to partial inhibition of retinal HMGCR activity (61, 62). This further verifies the local activity of the mevalonate pathway in the retina.

In another study, the de novo synthesis of both cholesterol and dolichol in *frog* retina was assessed using the same fundamental principles, but with two essential differences: the study was performed in vitro, rather than in vivo; and [^3^H]water (rather than [^2^H]water) was used, with separate, parallel incubations using [^3^H]acetate as the radiolabeled de novo precursor (63). The specific activity of radiolabeled products was determined by radio-HPLC. The majority of the [^3^H]acetate was incorporated into squalene, rather than into sterols; in addition, the frog retina was found to contain significant steady-state levels of squalene mass (unlike other vertebrate retinas). Hence, the flux of acetate into new cholesterol molecules was trapped in the squalene pool. The absolute rate of cholesterol synthesis was found to be only 3.4 pmol/h. This suggests that the de novo biosynthetic of sterol products in the retinas of amphibians (poikilotherms) are lower than those of warm-blooded species (homeotherms); hence, they cannot be compared directly with results obtained with rodent retinas. This was subsequently verified in vivo by intravitreal injection of [^3^H]acetate in frogs. Although acetyl CoA incorporation to mevalonate pathway is limited because of its hydrolysis (64), [^3^H]acetate was mostly incorporated into [^3^H]squalene, as well as [^3^H]cholesterol, detected both in whole retinas and in isolated rod OS membranes derived therefrom (65). Taken together, the in vitro and in vivo systems described above reflect squalene and sterol biosynthetic capacity in the neural retina of rodents and amphibians. It should be appreciated that these results apply to total retinal sterol synthesis but do not address or exclude the possibility that some portion of the sterols utilized by retinal neurons, including photoreceptor cells, may be derived from glia (i.e., Müller cells), as is the case in the CNS (66, 67). Future investigation of the mevalonate pathway in a retinal cell type–specific manner might provide additional critical insights into this aspect of retinal sterol homeostasis.

We now turn our discussion to the activity of postsqualene branch of the mevalonate pathway in the neural retina. Such investigations have involved the pharmacological targeting of the Kandutsch-Russell pathway or the Bloch pathway (see Fig. 2), followed by an assessment of the impact of such treatments on retinal structure and function. This mimics what occurs in some relatively rare hereditary disorders where the synthesis of cholesterol is decreased and its immediate precursor accumulates (19). A prime example of this is SLOS, the most common recessive disorder affecting the mevalonate pathway. The key biochemical signature of this disease is the accumulation of 7DHC in bodily tissues and fluids and clinically characterized by dysmorphologies, such as 2–3 toe syndactyly, craniofacial malformations, cognitive defects (autism spectrum), as well as rod and cone function deficits (68, 69, 70, 71). The latter point serves as *prima facie* evidence indicating a requirement for cholesterol to support normal retinal function (1). The mevalonate pathway intermediate 7DHC is highly prone to oxidation (72) and generates a spectrum of cytotoxic oxysterol metabolites, implicating them in the observed pathology (73, 74, 75). A pharmacological model of SLOS has been generated by systemic treatment of rats with AY9944 [(trans-1,4-bis(2-dichlorobenzylamino-ethyl)) cyclohexane dihydrochloride], a DHCR7 inhibitor (76). AY9944-treated rat retinas exhibit significant accumulation of 7DHC (7DHC/Chol mole ratio > 5) compared with control rat retinas (7DHC/Chol mole ratio < 0.1). Increased steady-state levels of 7DHC in the neural retina were associated with a progressive, caspase 3–independent, photoreceptor-specific retinal degeneration characterized by pyknotic (and TUNEL positive) photoreceptor nuclei, progressive shortening of rod OS, and thinning of the photoreceptor layer of the retina, defective clearance of shed rod OS tips by the RPE, and markedly decreased and delayed responses to light stimulation, indicating both rod and cone visual transduction defects (76, 77, 78).

Pharmacological targeting of DHCR7 also has been used to assess retinal cholesterol uptake. AY9944-treated Sprague-Dawley rats were maintained on a cholesterol-free diet until postnatal (PN) day 28 and then randomized into two dietary groups: one continued to receive a cholesterol-free diet, whereas the other was fed the same chow base, but supplemented with 2% (w/w) cholesterol, and then both groups were continued on this treatment until PN day 74 (79). 7DHC was the predominant sterol species in the retinas of AY9944-treated rats fed the cholesterol-free diet (7DHC/Chol mole ratio, 5.65), whereas cholesterol was the dominant sterol in retinas of those maintained on a cholesterol-enriched diet (7DHC/Chol mole ratio, 1.40). However, the *total* sterol content of the retina did not change appreciably under the conditions used, indicating that there was a 1:1 molar replacement of 7DHC by diet-derived, blood-borne cholesterol in the retina. (At the time, this was a striking finding because it had been assumed that the blood-retina barrier was similar to the blood-brain barrier in that it would exclude the uptake of blood-borne cholesterol.) The serum 7DHC/Chol mole ratio was 7.30 in the cholesterol-free dietary group and 0.10 in the cholesterol-enriched dietary group (79). These findings are in fair agreement with the above discussed metabolic approach using dietary [^2^H]cholesterol supplementation; both clearly demonstrate that blood-borne cholesterol is able to cross the blood-retina barrier and be taken up by the neural retina (60, 79). This has been verified through another independent approach, using intravenous injection of human LDL particles “doped” with cholestatrienol (cholesta-5,7,9(11)-trien-3β-ol), a naturally fluorescent derivative of cholesterol (80). The incorporation of cholestatrienol into the retina was followed as a function of the postinjection time, using confocal fluorescence microscopy; in parallel, rats were injected intravenously with human LDL particles containing [^2^H]cholesterol, and its uptake was followed by LC-MS analysis of retinal lipid extracts. Cholestatrienol fluorescence was observed in the choroid, RPE, and the distal outer neural retina within 2 h after injection of derivatized LDL; within 4 h, the entire outer retina fluoresced (including the photoreceptor cells), and by 6 h, the entire neural retina exhibited the brilliant blue fluorescence characteristic of UV-excited cholestatrienol. LC-MS confirmed the presence of [^2^H]cholesterol, in parallel, in the neural retina, increasing as a function of the post-injection time (80). Taken together, these independent lines of evidence demonstrate conclusively that cholesterol carried by LDL particles in the blood can be taken up by the retina and be broadly distributed throughout the neural retina (see also discussion below).

The previously discussed in vivo pharmacological and metabolic approaches have provided insights into cholesterol synthesis and uptake at the steady state in the mature neural retina, notably in rodents. However, the retina consists of both neuronal and glial cell types, arranged in discrete histological layers (see Fig. 1). An important question that remains to be investigated is the relative contributions of each of those cell types to overall retinal sterol homeostasis. The neural retina expresses the signature proteins involved in sterol synthesis and the lipoprotein uptake pathway. Quantitative PCR analysis has demonstrated the expression of presqualene and postsqualene enzymes, including MVK, HMGCR, DHCR24, and DHCR7 in the neural retina (62, 81). However, immunohistochemical analysis of murine retina revealed strongest expression of HMGCR in the *inner* retinal layers, rather than in the outer retina (where the photoreceptor cells reside) (62, 81). Correlative in situ hybridization analysis of key enzymes of the presqualene and postsqualene steps of the mevalonate pathway in other species may provide a morphological context to retinal cell type–specific differences in mevalonate pathway regulation. In this regard, a recent study performed single-cell RNA-Seq experiments on 90-day-old human retinal organoids derived from human embryonic stem cells (82). The human embryonic stem cells were genetically engineered to drive GFP expression under the control of the CRX promoter (cone-rod homeobox gene), which is expressed in developing precursor and mature photoreceptor cells, as well as inner retinal neurons such as CHX10-positive bipolar cells (83, 84, 85), to be able to selectively sort out expression of mevalonate pathway enzymes in CRX-GFP–positive retinal neurons cells versus other (GFP-negative) retinal cell types. HMGCR, SQLE, MVK, and other players of the mevalonate pathway were significantly enriched in the CRX-*negative* cluster, while GFP-positive cells (photoreceptors) only expressed basal levels of mevalonate pathway genes (82), possibly because of the requirement for de novo nonsterol isoprenoid synthesis, for example, dolichols (50). Furthermore, a recent in silico modeling of retinal sterol homeostasis suggests that photoreceptors acquire sterol from exogenous sources, rather than mevalonate pathway (86).

Hence, taken together, these results suggest that de novo synthesis of cholesterol in photoreceptors, per se, is likely minimal. This is curious and unexpected, given the prodigious daily rate of membrane assembly and turnover of photoreceptor OS membranes, which requires a continuous supply of lipids (including cholesterol) and proteins (14, 87). Also, it should be appreciated that a significant level of lipid (including cholesterol) synthesis is required during retinal development and maturation. Future investigations into retinal sterol homeostasis during retinal development may provide important insights into congenital disorders involving defective sterol homeostasis. Such investigations are now possible because of the recent development of a transgenic mouse line (mRX-Cre) that exhibits retina-specific Cre-recombinase expression (starting at day E8.5), driven by the Rx promoter (retina and anterior neural fold homeobox (RAX)), the earliest retinal determinant factor (88). Investigations into retinal neuronal- and glial-specific inhibition of the mevalonate pathway, using targeted gene-deletion methods, also should shed light on neuron-glial interactions in the retina that contribute to maintenance of retinal cholesterol homeostasis.

### Intraretinal cholesterol exchange: role of Müller glial cells

Intravitreal injection of radiolabeled amino acids, followed by tracking the fate of newly synthesized retinal proteins by light and electron microscopy-level autoradiography, first demonstrated the diurnal process of photoreceptor OS renewal and the intimate involvement of RPE cells in this process (14, 87). A similar approach was adapted to demonstrate the continual synthesis and distribution of APO-E, a signature protein associated with VLDL and IDL particles, in the rabbit neural retina, and in a primary Müller glial cell culture model (89). Radiolabeled amino acids were faithfully incorporated into APO-E within 3–6 h after intravitreal injection. SDS-PAGE autoradiography of immunoprecipitated APO-E showed its presence mostly in the vitreous and in the neural retina, with little incorporation in the optic nerve (89). This agrees with the results of neuron-glia coculture studies, which showed that cholesterol, but not APO-E, is required for retinal ganglion cell synaptogenesis (66, 67). Transcriptomic analysis of purified mouse Müller glial cells and in situ hybridization of mouse retinal tissue sections also demonstrated APO-E expression in Müller glia, along with other classic markers, such as aquaporin-4, RLBP1, and GLUL (90). Additional evidence shows synthesis and export of APO-E– and APO-J–containing lipoproteins, varying in density from 1.006 to 1.180 g/cc and diameters of 14–45 nm, in primary rabbit Müller glia (91). This is consistent with the results of studies using CNS-derived astrocytes, which also secrete APO-E and APO-J and are thought to play a role in retinal sterol homeostasis (89, 91, 92). Thus, Müller glia have the capacity to assemble and secrete lipoproteins or lipoprotein-like particles, which then can be utilized by adjacent cells (e.g., photoreceptors or inner retinal neurons), and may serve as a local, intraretinal source of cholesterol. Retinas from APO-E–null mice have been reported to have a significant increase (2.8-fold) in their unesterified cholesterol content, and this increase was observed despite a compensatory increase in retinal APO-B levels. This suggests an important role for retinal APO-E synthesis for tissue sterol redistribution (93). The requirement of Müller cells in retinal cholesterol homeostasis could be validated by cell type–specific ablation of genes coding for postsqualene pathway enzymes; for example, breeding mice harboring floxed mevalonate pathway genes with mice expressing Cre recombinase under the control of a Müller cell–specific promoter (94, 95).

A study using APO-E knockout mice reported electrophysiological deficits, accompanied by possible dropout of Müller glial cells, by PN 25 weeks of age (96). In another study, the lack of APO-E also reportedly lead to an appreciable (3-fold) increase in retinal cholesterol content compared with age-matched controls, with the majority of the total retinal cholesterol being unesterified (93). (We have not been able to reproduce those findings independently; c.f., Fliesler, S.J., M.J. Richards, N.S. Peachey, K. Kauser. Hypercholesterolemia does not alter retinal sterol composition or compromise retinal structure or function in APO-E–knockout mice. *Invest. Ophthalmol. Vis. Sci. (ARVO Abstr.)* 2000; 41:S199.) Also, a compensatory increase in the retinal expression of other lipoproteins, such as APO-A4 and APO-B, was observed upon APO-E gene ablation (93).

APO receptors also play an important role in retinal development. Undifferentiated neuronal precursors express APO-A1 and the SRB-I (97). Rod photoreceptor differentiation, maturation, and synaptogenesis temporally coincide with Müller glial development and their synthesis of APO-E (97). In the CNS, a major receptor for APO-E is LDL-related particle 1 (LRP1), which is required for synaptogenesis, oligodendrocyte progenitor cell differentiation, and myelination (98, 99). LRP1 is expressed in primary RPE cells (100), as well as in retinal endothelial and Müller glial cells (101, 102). However, the specific requirement of LRP1 for sterol homeostasis in retinal neuronal cell types remains to be assessed. Taken together, the evidence extant suggests a role for APO secretion by Müller glia in maintaining cholesterol homeostasis in the neural retina. *In vivo* evaluation of neuronal uptake of Müller glia–derived sterols by surrounding neurons is not possible using conventional metabolic approaches. This is due to the inability to “tag” the de novo–synthesized sterol with a fluor and follow its trafficking, secretion and uptake because sterols (unlike proteins) are not coded by genes. However, an alternative approach might be targeted deletion of enzymes of the postsqualene pathway in Müller glia, such as sterol-C5-desaturase, DHCR24, or DHCR7, and follow-up assessment of uptake and incorporation of the biogenic cholesterol precursor into neighboring retinal neurons (e.g., photoreceptor cells) (see Fig. 3).

### Mevalonate pathway activity in the RPE

The above results pertain only to de novo cholesterol synthesis and uptake in the *neural* retina. We will now specifically consider the mevalonate pathway in the RPE. Although immunohistochemical analysis has shown the presence of HMGCR in human and murine RPE cells (62, 81), investigating RPE cholesterol synthesis rates in vivo is extremely challenging because of the technical difficulties involved in the metabolic approaches and the need for targeted cell type–specific inhibition of the mevalonate pathway. As a potentially more tractable and fruitful alternative, RPE in vitro models of genetic diseases pertaining to the mevalonate pathway and related pharmacological models as well as the conventional metabolic approach may be of utility to investigate RPE de novo sterol synthesis. Recently, we generated a human induced pluripotent stem cell (iPSC)–derived RPE in vitro model of SLOS (point mutations in *DHCR7*, leading to hampered reduction of 7DHC to cholesterol), comparing SLOS RPE cells (generated from iPSCs from fibroblasts isolated from patients with well-characterized SLOS) with iPSC-derived RPE cells from normal human controls (77). SLOS-RPE cells cultured in delipidated serum showed elevated steady-state levels of 7DHC (∼40% of total sterol content), unlike the control RPE cells (which had minimal 7DHC content), indicating an active cholesterol synthesis pathway (77). Other studies have demonstrated in vitro incorporation of radiolabeled acetate into cholesterol in ARPE-19 cells, an immortalized human RPE–derived cell line (103). *In vivo* study utilizing RNASeq analysis suggest that diurnal changes occur in the expression of mevalonate pathway genes, such as *Hmgcr*, *Dhcr24*, and *Sqle*, in the RPE of 10- to 13-week-old mice (104). These results qualitatively demonstrate the ability of RPE cells to synthesize cholesterol autonomously but do not permit the calculation of absolute sterol synthetic rates.

### Role for RPE in retinal cholesterol uptake

Uptake of blood-borne LDL particles by the RPE was first demonstrated utilizing tail vein injection of Rhodamine-labeled LDL particles and subsequent monitoring of their uptake by the RPE using fluorescence microscopy (105). However, fluorescent tags are not ideal tracers because their conjugation to LDL may be unstable in vivo. *In vitro* experiments utilizing immortalized ARPE-19 cells demonstrated LDL receptor (LDLR)-mediated uptake of LDL (105). As described above, RPE-mediated uptake of circulating, LDL-bound cholesterol was demonstrated in rats using intravenous injection of *human* LDL equilibrated with unesterified cholestatrienol, monitoring the incorporation of the fluorescent sterol by confocal fluorescence microscopy (80). In that study, LDL uptake also was monitored by immunohistochemistry and correlative Western blot analysis of the neural retina with a monospecific antibody against human APO-B (importantly, with no cross-reactivity against rat APO-B). Immunohistochemistry confirmed the presence of human APO-B immunoreactivity in the RPE and the neural retina, whereas Western blot analysis showed an immunoreactive 80-kDa APO-B peptide (consistent with partially degraded human APO-B), indicating endocytic uptake and degradation of LDL particles by the RPE and the neural retina (80, 106). On the other hand, serum deprivation of ARPE-19 cells has been shown to result in transcriptional upregulation of mevalonate pathway genes, as well as accumulation of cellular free cholesterol (107). LDLR expression has been observed in the RPE, as well as in the ganglion cell layer (GCL) and in the endothelial cells of the retinal vasculature in human and monkey retinas (62, 80, 81). In another study, LDLR knockout mice were generated on an ApoB100 background (ApoB100 LDLR^−/−^), and their retinas were probed with filipin, a naturally fluorescent macrolide antibiotic molecule that binds to the 3β-hydroxyl group of sterols, to localize cholesterol in various cell types (108). ApoB100 LDLR^−/−^ mice exhibited deficits in scotopic (rod-driven) electroretinogram (ERG) responses, as well as accumulation of esterified cholesterol on the basolateral surface of the RPE, consistent with inefficient uptake of LDL particles (108). The acid lipase-dependent processing of endocytosed lipoproteins in RPE cells is sensitive to buildup of bisretinoid adducts such as A2E, which displaces cholesterol from lipid rafts (109). Taken together, the results suggest the involvement of LDLR-mediated uptake of LDL by the RPE and the neural retina, the subsequent endolysosomal processing of LDL, and ultimate incorporation of LDL-derived free cholesterol into the neural retina.

Mutations in the *NPC-I* gene lead to accumulation of free cholesterol in lysosomes because of deficient NPC-I–mediated transfer of free cholesterol from lysosomes to the ER (110). In a genetic mouse model of NPC-I disease, significant accumulation of free cholesterol (determined by filipin binding) was observed in the RPE and in the outer plexiform layer of the retina (111). Also, retinal degeneration is observed in NPC-I–modified genetic mouse models, characterized by an age-dependent decrease in dark-adapted (rod-driven) a-wave and b-wave ERG responses, as well as by progressive, irreversible photoreceptor-specific cell death (observed as exclusive TUNEL -positive labeling in the outer nuclear layer) (111, 112). This animal model further exhibited defective autophagy, accumulation of membranous and lipid inclusion bodies, aberrant dendritic arborization, and neurite stratification defects in retinal neurons, but without increased filipin staining in retinal neurons (112). Spectral domain-optical coherence tomography imaging of the neural retina of NPC-I patients has revealed significant thinning of the nerve fiber layer and axonal degeneration (113). Surprisingly, however, patients with LAL deficiency, which results in accumulation of lysosomal cholesteryl esters, do not exhibit retinopathies (114). Although LAL is required for RPE cholesterol homoeostasis (115), the effect of conditional deletion of LAL in retinal neurons remains to be directly investigated. Overall, these defects reflect a neuronal requirement for glia-derived cholesterol in the retina, as discussed earlier.

Cholesterol uptake also occurs through SRB-I and SRB-II, which are involved in the uptake of HDL and OxLDL, respectively. Binding studies using [^125^I]-labeled LDL and acetylated-LDL particles and bovine RPE cells have demonstrated that RPE cells possess both LDLR and scavenger receptor activity (116). When challenged with excess unlabeled LDL or acetylated LDL in vitro, cultured bovine RPE cells responded by downregulating the LDLR, but not scavenger receptors, which is typical of macrophages and arterial endothelial cells. Primary human RPE cells also have been shown to express SRBs (117). The transcript and protein level expression of SRB-I/SRB-II receptors in primary RPE cells was examined using RT-PCR, and by analyzing incorporation of radiolabeled amino acids into newly synthesized SRB-I (117). A comprehensive study showed the differential expression of APOs and SRB reporters in the monkey retina. Although both the RPE and the GCL showed APO-A1, SRB-I, and SRB-II immunoreactivity, photoreceptors expressed only class B scavenger reporters (80). These findings suggest a role for complex intraretinal lipoprotein transport mechanisms in maintaining sterol homeostasis in the neural retina and in the RPE (80, 117).

SRB-I is involved in cellular uptake of cholesteryl esters from HDL particles, as well as lutein uptake by the RPE (118). SRB-I also participates in cholesterol efflux from extrahepatic tissues, thereby performing both uptake and efflux roles (119). Further understanding of SRB-I requirement for proper retinal functioning has been achieved using a global SRB-I knockout model (120). This model exhibits hypolipoproteinemia and concurrent increase in serum cholesterol levels, which is further exacerbated by feeding a high-fat diet. SRB-I knockout mice fed a normal chow diet exhibited mild decreases in dark-adapted a-wave and b-wave ERG responses, as compared with controls, but without any observable retinal structure abnormalities (120). However, those mice fed a high-fat diet exhibited photoreceptor layer disorganization, sparse sub-RPE lipid deposits, ERG abnormalities, and significant thickening of Bruch's membrane (the extracellular matrix interface between the choriocapillaris and the RPE (120)).

### Sequestration of cholesterol in storage depots

A key mechanism in cellular cholesterol homeostasis is the esterification of excess free cholesterol and its storage in lipid droplets (121, 122). Three enzymes catalyze the esterification of ACAT1 and ACAT2 and LCAT (123). Both human and macaque retinas express ACAT1 and LCAT (80, 124). ACAT1 immunolocalization also has been reported in the murine retina in the photoreceptor OS layer, outer plexiform layer, GCL, and in the RPE (81). However, LCAT appears to localize to Müller glial cells, rather than to retinal neurons (125). Also, LCAT ablation does not lead to retinal degeneration or dysfunction (126). Inhibition of cholesterol efflux, such as that observed in CYP27A1/46A1 double-knockout mice, with resultant increase in the photoreceptor cholesterol content, exhibits ACAT1-dependent esterification of sterols in photoreceptors, notably in their OSs (125). The latter finding is curious, considering the fact that, historically, extensive lipid composition analyses across multiple vertebrate species have failed to detect such molecules in purified photoreceptor OS membrane preparations (12).

## Cholesterol efflux in the neural retina and RPE

### Role of ABC transporters in retinal cholesterol efflux

ABC transporters ABCA1 and ABCG1 generally account for the majority of cellular sterol efflux, depending on tissue expression levels (25). ABCA1 and ABCG1 are expressed widely in most tissues, including brain, retina, and macrophages (32), whereas ABCG4 is predominantly expressed in the brain (127). ABCG1 additionally caters to efflux of sterols derived from OxLDL to HDL particles in macrophages, and plays a protective role in atherosclerosis (128). ABCG1 is expressed in the developing and the mature retina (32, 127). Brain tissue from *Abcg1-Abcg4* double-knockout mice show significant accumulation cholesterol and lathosterol (cholest-7-en-3β-ol), as well as 24-OH-Chol, 25-OH-Chol, and 27-OH-Chol by 8 months of age, compared with age-matched controls. Therefore, ABCG1 and ABCG4 play a key role in brain sterol efflux (127). ABCG1 and ABCG4 are expressed in all the layers of the neural retina and in the RPE, as well as in primary cultures of Müller glial cells and ganglion cells (32, 127, 129). Retinal histological maturation appears normal in *Abcg1-Abcg4* double-knockout mice, which exhibit mild retinal dysfunction accompanied by a relatively small increase in lathosterol content (unlike the accumulation of oxysterols and cholesterol in the brain) (127).

ABCA1 and ABCG1 expression in the neural retina increased upon treatment with the LXR-α agonist T0901317, suggesting a role for both LXR-α regulation and ABC transporters in retinal sterol efflux (62). Rod photoreceptor–specific knockout of ABCA1, using Rho-iCre mice (130), leads to appreciable lipid droplet accumulation and age-related retinal dysfunction at around PN 12 months (131). Rod photoreceptor–specific ABCA1/G1 knockout (unlike ABCG1/G4 knockout) leads to increased levels of retinal cholesterol, 7KChol, and 24-, 25-, and 27-OH-Chol by PN 12 months (131). Maintaining rod-specific ABCA1/G1 knockouts on a high-fat diet accelerates lipid accumulation and retinal degeneration (131). Similar age-related retinal dysfunction and cholesterol accumulation (around PN 10–14 months) occur upon LXR-α deletion, with subsequent reduction in ABC transporter levels (132). By contrast, LXR-β knockout did not lead to photoreceptor dysfunction (even up to PN 10 months). However, a lack of LXR-α and/or LXR-β leads to lipid accumulation in the RPE (132). Also, LXR-β knockout causes slow, progressive loss of ganglion cells over the course of PN 18 months, accompanied by decreased retinal aquaporin-4 expression as well as microglial activation (indicative of neuroinflammation) (133). Curiously, the onset of retinal dysfunction upon inhibition of cholesterol efflux at the level of LXR-α/β, or ABC transporters (also CYP hydroxylase knockouts, discussed later) leads to a slow retinal degeneration phenotype that manifests by about 1 year. This is unlike inhibition of retinal de novo cholesterol biosynthesis either through statin treatment (which most likely is due to protein prenylation compromise, rather than blockade of sterol synthesis) or as observed in the AY9944-induced model of SLOS (see below), which leads to retinal degeneration on a timescale of weeks (76, 131, 132, 133). In sum, these observations independently indicate significant differences in the rates of retinal sterol synthesis and turnover, in agreement with the results of previous studies (60, 79).

### Cholesterol efflux in the RPE

ABCA1 and ABCG1 expression has been reported for the RPE, in addition to the neural retina (81, 129, 134). Human and murine RPE/choroid express all of the known components of the cholesterol efflux mechanism (LXR-α/β, ABC transporters, APO-A1, APO-E, APO-B) as well as players in intracellular sterol transport (NPC-I, translocator protein 18 kDa [TSPO]) (135). Native RPE cells and ARPE-19 cells express microsomal triglyceride transfer protein and APO-B, suggesting their capability to assemble their own lipoprotein-like particles (presumably for export) (124). ARPE-19 cells cultured with [^3^H]oleate have been show to secrete [^3^H]-labeled cholesteryl esters and triglycerides into the culture medium, and the lipoprotein-like particles isolated from the culture medium had physical characteristics (e.g., d << 1.21 g/ml) comparable with plasma lipoproteins (124). APO-A1 expression has been documented in the human and monkey RPE, as well as in the neural retina (80, 97, 136, 137). RPE-specific double knockout of ABCA1 and ABCG1 leads to accumulation of cholesteryl ester–rich lipid droplets in the RPE, accompanied by frank degeneration of the neural retina by PN 6 months (134). Furthermore, RPE-specific ABCA1 knockout was sufficient to cause lipid droplet accumulation, suggesting an important role for ABCA1 in RPE cholesterol efflux (134). ABCA1-dependent cholesterol efflux in the RPE is sensitive to treatment with probucol (a potent *bis*-phenol antioxidant that also inhibits ABCA1) and ABCA1 antibodies (135, 138). Poorly polarized ARPE-19 cells fail to stimulate basolateral cholesterol efflux to APO-A1, unlike well-differentiated and polarized human primary RPE cells (with healthy transepithelial electrical resistance). Polarized RPE can engage in ABCA1-mediated sterol transfer to APO-A1 on both the apical and basolateral sides of the cell (135). Furthermore, RPE cells can successfully efflux cholesterol derived from photoreceptor OS membranes on both the apical and basolateral side of polarized RPE in an APO-A1–dependent manner (135). It is important to meet the following criteria when using primary, iPSC-derived, or transformed RPE cells for these kinds of studies: (1) the cells should be well polarized (i.e., have defined apical and basolateral compartments); (2) the culture medium should have a defined lipid content (as well as the lactate content); (3) cells should have proper *trans*-epithelial electrical resistance; (4) the cytoskeleton and microtubule alignments should be comparable with those observed in normal RPE cells in vivo; (5) the genotype should be confirmed when using human donor primary or iPSC-derived cells; and (6) differences in lipid-related genes should be documented between the various models used (139, 140).

Conditional ablation of *Abca1* and *Abcg1* in macrophages leads to thickening of Bruch's membrane and lipid droplet accumulation in the RPE as well as in the subretinal space (141). Under these conditions, both esterified and unesterified cholesterol content increase in the retina and RPE/choroid, as compared with age-matched controls (141). Using this type of model, concomitant age-related retinal degeneration was observed, as characterized by decreased ERG responses and the appearance of macrophages in the subretinal space and choroid by PN 12 months. These are common features observed in several retinal degeneration animal (primarily mouse) models, as well as in human AMD (141, 142, 143, 144). Collectively, these findings suggest a role for macrophage interactions with the RPE in efficient cholesterol efflux across the outer blood-retinal barrier.

### CYP enzyme-catalyzed sterol hydroxylation and oxidation in the neural retina

Two CYP genes are expressed in the neural retina: CYP27A1 and CYP46A1. The oxidized cholesterol derivatives 27-COOH-Chol and 27-OH-Chol (metabolites of CYP27A1) are the predominant oxysterol species found in human and bovine retinas, which stimulate LXRα-dependent cholesterol efflux (145). CYP27A1 expression was observed in ARPE-19 cells, as well as photoreceptor ISs, ganglion cells, and RPE of the monkey retina (146). 27OH-7KCh, a product of CYP27A1-mediated metabolism of 7KChol, was found to be significantly less cytotoxic to ARPE-19 cells than 7KChol (146). Under conditions of elevated oxidative stress and lipid peroxidation, CYP27A1 undergoes modification by lipid peroxide products, such as isolevuglandins, leading to reduced enzymatic activity, in turn contributing to altered cholesterol homeostasis (147).

TSPO is a transmembrane protein involved in translocation of cholesterol from the outer to the inner mitochondrial membrane (148, 149). Thereby, TSPO regulates sterol substrate availability to inner mitochondrial membrane resident CYP27A1 and plays a regulatory role in sterol efflux (150). Activating ligands of TSPO, such as FGIN-1-27, increase RPE cholesterol efflux and decrease cellular cholesterol and phospholipid levels (103). TSPO knockdown sensitizes ARPE-19 cells to OxLDL challenge, leading to increased reactive oxgen species (ROS) generation and expression of inflammatory cytokines, such as interleukin-1β and TNF-α (103). Immunohistochemical analysis suggests expression of TSPO in the RPE and GCLs of the mouse retina. RPE TSPO expression levels decline with age and correlate with accumulation of cholesterol in the cell (103). The increase in RPE ROS levels also is accompanied by increase in the GSSG:GSH ratio (an indicator of oxidative stress), accumulation of free fatty acids, and decreased cellular ATP and NADH content (151).

Other CYP enzymes involved in lipid efflux also affect cellular cholesterol homeostasis. For example, cholesteryl ester-laden lipid droplet accumulation and autophagic defects also have been observed in an iPSC-derived RPE model of Bietti's crystalline dystrophy, which is caused by mutations in the gene encoding CYP4V2 (152, 153). CYP4V2 is required for ω-oxidation of fatty acids, and the RPE lipid accumulation observed in the Bietti's crystalline dystrophy in vitro model was partially relieved by cyclodextrin treatment (153). This finding suggests a role for CYP hydroxylase–mediated fatty acid oxidation in RPE lipid efflux.

ER-resident cholesterol-24S-hydroxylase (CYP46A1), which catalyzes the rate-limiting step in brain cholesterol efflux, metabolizes cholesterol to 24S-hydroxycholesterol (24S-OH-Chol) (154, 155, 156). In the retina, CYP46A1 is expressed predominantly in the inner retinal layers and in the RPE but is comparatively low in the photoreceptor layer (157). Intravitreal injection of albino rats with voriconazole, a CYP46A1 inhibitor, did not lead to retinal degeneration or altered dark-adapted ERG responses (158). However, intraperitoneal injection of voriconazole led to a significant decrease in retinal 24S-OH-Chol levels without concomitant changes in brain or serum levels within 5 days (159). This is very surprising, given that CYP46A1-synthesized 24S-OH-Chol is the predominant cholesterol elimination product in the brain, unlike the retina, which strongly depends on CYP27A1-dependent metabolism for cholesterol efflux (154, 155, 160, 161). A global knockout mouse model of CYP46A1 exhibited a significant, compensatory increase in retinal cholestenoic acid (a by-product of 27-OH-Chol oxidation by CYP27A1), and subsequent activation of LXRα/β and their gene targets (162). However, no significant changes in retina cholesterol content or ERG rod- or cone-driven responses were observed in 6-month-old CYP46A1 knockout mice compared with age-matched controls at PN 6 months (162). The retinas of CYP46A1 knockout mice exhibited leaky vasculature and microglial activation (162).

CYP27A1/CYP46A1 global double-knockout mice (*CYP27A1*^−/−^-*CYP46A1*^−/−^) exhibit elevation in retinal cholesteryl ester content in lipid droplets. As discussed above, remarkably, such lipid droplet accumulation was observed in the OS layer (125). The total cholesteryl ester and 7KChol content was significantly elevated in the retina, liver, brain, and lungs of the double-knockout mice (125, 163), which also exhibited aberrant angiogenesis and retinal vasculature defects (163). The accumulation of retinal cholesteryl esters was fully inhibited by deletion of *Acat1* (required for sterol esterification) on the double-knockout background (125). The sterol profile in triple-knockout (*CYP27A1*^−/−^-*CYP46A1*^−/−^-*ACAT1*^−/−^) mice was comparable with that of controls. (125, 162). Despite normalization of the sterol profile, photoreceptor cell death was observed (using TUNEL labeling) (125, 162). Future investigations into ABC transporter activity in *CYP27A1*^−/−^-*CYP46A1*^−/−^-*ACAT1*^−/−^ and *CYP27A1*^−/−^-*CYP46A1*^−/−^ models may provide additional new insights into the retinal cholesterol efflux mechanism. The accumulation of cholesteryl esters in the retina and RPE in the above discussed animal models has been observed using Oil Red O staining, filipin labeling, and fluorescence imaging of retinal tissue sections (plus and minus cholesteryl esterase treatment), as well as transmission electron microscopy (125, 134, 163). These methods along with lipidomic analysis of retinal tissue to quantify total and free sterol (and, by difference, esterified cholesterol) have provided compelling validation of retinal sterol esterification (163).

## Ox-Ldl and oxysterols on the neural retina and the RPE

We have previously discussed the critical role oxysterols play as LXR agonists, thus facilitating ABC transporter–mediated cellular cholesterol efflux. Therefore, oxysterols act as regulators of cholesterol biosynthesis and metabolism (164, 165). Elevated oxysterol levels in membranes lead to organelle dysfunctions such as mitochondrial dysfunction, ER stress, and lysosomal membrane permeabilization (166). High-intensity light exposure leads to nonenzymatic lipid peroxidation and generation of 4-hydroxynonenal (4-HNE, a by-product of oxidation of *omega*-6 PUFAs) and, subsequently, oxidatively modified retinal proteins (167). Similar conditions can promote the nonenzymatic oxidation of cholesterol and other sterols, generating cytotoxic oxysterols (168, 169, 170, 171, 172). In addition to conditions that cause photo-oxidative stress, alterations in iron homeostasis also have been shown to promote lipid peroxidation (173, 174), including in the retina (175, 176). The chemistry of oxysterols and their biophysical effects on the plasma membrane have been discussed above, as well as in other reviews (177, 178). We will now discuss the uptake mechanisms of OxLDL and the biological effects of oxysterols and OxLDL in the RPE and the retina.

### OxLDL and oxysterols in the RPE

The RPE cell is a unique epithelial cell type because it is a postmitotic, long-lived professional, stationary phagocyte, while also possessing the classic characteristics of a polarized epithelial cell and participating in barrier functions like other epithelial cells. Key insights into RPE cholesterol homeostasis have arisen from studies performed using in vitro models, given the ease of establishment and long-term maintenance of primary RPE cultures, availability of transformed RPE-derived cell lines (RPE-J, ARPE-19, etc.), and recent advancements in iPSC-derived RPE in vitro cell models (140, 179, 180). However, it should be kept in mind that ARPE-19 cells, in addition to be immortalized, are not fully differentiated and have some distinct differences from primary RPE cells that may limit the direct applicability of results obtained with their use to the normal biology of the RPE (140, 180). However, in vitro oxysterol treatment leads to direct free oxysterol incorporation into the plasma membrane or entry into the cell, by-passing the canonical endocytic uptake pathway.

Uptake of OxLDL occurs primarily via a receptor-mediated endocytic uptake pathway dependent on either SRB CD36 or the lectin-like LOX-1 (105). In the retina, a primary function of SRB-II is its role in the uptake of shed photoreceptor OS tips by the RPE during the daily process of photoreceptor membrane turnover (181). Expression of SRB-II has been observed in primary RPE cells, ARPE-19 cells, and in animal models (80, 105, 117). Both CD36 and internalize various cargoes, such as cholesteryl esters and phosphatidylserine-rich membranes. RPE cells exhibit CD36-dependent phagocytic uptake of OS phospholipids (181, 182, 183). On the other hand, the lipid peroxide species generated by photo-oxidation of OS membranes (e.g., of their constituent PUFA-containing phospholipids, as well as sterols) serve as potent ligands for the CD36-mediated diurnal uptake of OS by the RPE (184). Furthermore, OxLDL and lipid peroxide products competitively inhibit CD36-mediated uptake of OS by RPE cells (184), and uptake of OxLDL mediated by CD36 is also blocked by antibodies against CD36 (185).

Treatment of RPE-J cells with OxLDL or oxidized OS membranes significantly decreased the degradation of phagocytosed OS by RPE cells (186). This was due to inefficient phagosome maturation observed as a lack of co-compartmentalization of opsin (the visual pigment apoprotein) with markers of the endolysosomal system, such as Cathepsin-D or Rab5 (186, 187, 188).

Primary RPE and transformed ARPE-19 cells challenged with OxLDL (100 μg/ml) undergo cell death, with transformed RPE cells exhibiting sensitivity at lower concentrations (189). RPE cell death was accompanied by elevated expression of vascular endothelial growth factor (VEGF), proapoptotic mediators like Bax (causes cell death upon mitochondrial membrane permeabilization), and generation of ROS (190, 191, 192, 193, 194, 195, 196, 197). Uptake of OxLDL by CD36 causes disruption of RPE barrier function, activation of the NRLP3 inflammasome complex, increased cellular cholesterol level, and accumulation of cholesteryl esters (138, 185). Similar to OxLDL, treatment of transformed and primary RPE cells with 7KChol and 25-OH-Chol led to cell death because of ROS generation (193, 194, 198, 199). Oxysterol treatment also caused upregulation of proinflammatory cytokine expression (i.e., IL-1B, IL-6, IL-8, and IL-18) and increased ABCA1-dependent efflux (194, 196, 198, 200, 201, 202, 203). Sterculic acid, a naturally occurring monounsaturated fatty acid, alleviated 7KChol-induced VEGF and interleukin expression (197). Oxysterols such as 7KChol trigger inflammatory responses and may therefore contribute to RPE and retinal pathology (198, 204).

Cholesterol homeostasis of the outer retina, especially as pertains to sterol and lipid uptake and efflux across the RPE, plays a critical role in the pathogenesis of AMD (86, 205). While rodent models serve as excellent biochemical systems to investigate sterol homeostasis and related monogenic disorders, the notable lack of a cone-rich fovea in rodents and other nonprimate species presents a challenge in reliable modeling AMD in laboratory animals. Key insights into the pathogenesis of AMD arises from lipidomic and proteomic analysis of drusen deposits obtained from human AMD patient donor eyes (206). The major lipid constituents of sub-RPE drusen deposits include esterified cholesterol, sphingomyelin, and phosphatidylcholine (206, 207). The fatty acid profile of lipoproteins isolated from drusen is rich in linoleate (a signature of blood-borne lipoproteins, influenced by diet), rather than DHA (a fatty acid highly enriched in photoreceptor OS membranes, but typically found in low levels in plasma), suggesting predominantly an extraretinal (systemic) origin (208). Furthermore, recent in vitro studies involving long-term primary porcine or human RPE cell cultures have shown the presence of drusen-like basolateral deposits, highlighting the endogenous capacity of RPE cells to generate and export such lipid-rich material (139, 209). These results are consistent with the proposed role for RPE sterol efflux in AMD pathogenesis. 7KChol levels in RPE-choroid of primates and humans increase with age and is the major oxysterol constituent of drusen (196, 210). Understanding the origins of the drusen lipid content has been aided by analysis of the accompanying proteome (206). Major peptide constituents of drusen include APO-E, APO-B, serum albumin (arising from blood), and also proteins possibly of RPE/retinal origin, such as complement factors (CFH, C3, C5), TIMP3, crystallins, and APO-A1 (206, 211). These lipidomic and proteomic findings suggest that serum LDL and outer retina sterol efflux both contribute to the formation of drusen deposits. The drusen proteome also was found to contain lipid peroxide adducts, suggesting the involvement of oxidative stress in drusen formation (211, 212, 213). Although a recent large-scale study suggests a lack of correlation between serum OxLDL levels of patients with AMD and drusen formation (214), this cannot rule out a critical role for retinal oxidative biology in AMD pathogenesis. This is because simple ELISA assays to quantify serum OxLDL levels do not detect individual oxysterol species synthesized locally due to chronic oxidative stress in the retina (213, 215). The latter is clearly evidenced by the accumulation of 7KChol and lipid peroxide adducts detected in drusen, and from the described biological effects of oxysterols on the RPE in vivo. These findings suggest that the oxysterols observed in drusen may arise from lipid peroxidation of already-formed drusen, rather than deposition of OxLDL.

RPE cells challenged with A2E (a bisretinoid by-product of the visual cycle) formed by condensation of phosphatidylethanolamine with two molecules of all-*trans* retinaldehyde derived from OS membranes (216) exhibit elevated levels of cholesterol oxidation. A2E displaces cholesterol from lipid rafts, thereby disrupting lipid rafts in RPE cells (109). A2E-induced accumulation of free cholesterol in the RPE endolysosomal system inhibits autophagic flux in the RPE (217). A2E and OxLDL treatment leads to increased tubulin acetylation, inhibiting retrograde phagosome trafficking (217, 218). Stimulation of RPE cholesterol efflux by transcriptional upregulation of ABCA1 and ABCG1 (using the LXR agonist T0901317) relieved A2E-induced deficits in phagosome maturation (217). Cholesterol is required for phagosome retrograde trafficking (219, 220). Phagosome maturation defects were observed in an iPSC-derived RPE in vitro model of SLOS, characterized by increase in cellular 7DHC levels (77). The sluggish degradation of phagocytized OS observed in the SLOS RPE model is also seen in genetic and pharmacological animal models of SLOS (77). The RPE in SLOS animal models also accumulates undigested OS and lipid droplets and exhibits increased lipofuscin and A2E content (76, 77). It should be noted that in SLOS in vivo models, sterol homeostasis in the RPE is altered both by the de novo synthesis of 7DHC and 7DHC-derived oxysterols and their uptake via receptor-mediated endocytosis of LDL and OxLDL. However, rodent and in vitro experiments investigating the effects of A2E on RPE have made two fundamental assumptions (1) that the RPE is homogeneous across the eye and (2) that A2E is homogeneously distributed across the eye, hence the presumption that data obtained from whole-eyecup cell isolations and lipid extractions are meaningful. These conditions may be true for rodents (221), but likely not for human and other foveated species (221, 222, 223, 224). Recently, foveal RPE cells isolated from human eyes have been determined to have unique properties compared with nonfoveal RPE cells (225, 226).

### OxLDL and oxysterols in the neural retina

Lipid peroxidation in rod OS membranes occurs nonenzymatically by formation of free radicals through Fe^2+^-mediated Fenton reaction (227). *In vitro* assays have demonstrated that light-induced lipid peroxidation of OS membranes may be inhibited by iron chelators, suggesting a role for Fe^2+^ (e.g., as derived from ferritin, in vivo) in such oxidation (228). *In vivo* administration of iron chelators significantly diminished retinal degeneration observed under conditions of intense light exposure in the “retinal light damage” model (229), as well as in some genetic models of retinopathies (230, 231, 232, 233). The role of iron homeostasis in the neural retina and in retinal pathologies has been discussed in depth elsewhere (234). Peroxidation and cyclo-oxidation of n-PUFAs (such as DHA and arachidonic acid) generate highly reactive α,β-unsaturated aldehydes (such as 4-HNE and carboxyethylpyrrole) and γ-ketoaldehydes (e.g., isolevuglandins and levuglandins) (147, 167, 235, 236, 237). These reactive aldehydes further form adducts with lysine, cysteine, and histidine residues of proteins in animal models of retinal light damage (235, 238). Transducin-α, a key component of the phototransduction cascade in photoreceptors, has been found to be adducted with 4-HNE in light damage model, implicating lipid peroxidation–mediated disruption of phototransduction in the observed retinal degeneration (235). Also, 4-HNE–modified proteins have been identified in retinas from a rat model of SLOS (239). Isolevuglandin modification of CYP27A1 also has been observed in a mouse model of retinal light damage (147), which was inhibited by pretreatment with pyridoxamine (a scavenger for γ-ketoaldehydes) (240). The structural and functional rescue of the photodamaged retina upon pyridoxamine treatment remains to be determined. The above studies together demonstrate that iron-mediated lipid peroxide modification of proteins is a critical component of the retinal pathology induced by bright light exposure. These findings underscore a role for lipid peroxidation in the retina upon elevated photo-oxidative stress, such as that observed in rodent models of retinal degeneration (241, 242, 243). However, such rodent model studies have not addressed the potential role of chronic normal (ambient) light exposure or how retinal photo-oxidative stress leads to AMD progression in humans (244, 245, 246, 247).

7KChol has been implicated in several age-related disorders (45, 165, 248). The predominant oxysterol found in the OxLDL formed in vivo (generated by copper- or iron-catalyzed oxidation of LDL) is 7KChol; other significant oxysterols include 7αβ-OH-Chol and 5,6α/β-epoxy-Chol (249). *In vitro* studies on the effects of exogenous OxLDL use copper- or iron-catalyzed oxidation of LDL to generate OxLDL. The types of oxysterols found in the neural retina of albino rats subjected to bright light conditions suggest involvement of the Fenton reaction in their formation (249). By contrast, oxysterol levels are minimal in control animals (not subjected to intense light exposure) and are attributable to basal levels of enzymatic (e.g., CYP27A1) and/or nonenzymatic sterol oxidation. The immunolocalization of 7KChol in photodamaged retinas is qualitatively comparable to that of H- and L-ferritin (a local, endogenous source of ferrous ions) in the neural retina, that is, the IS, inner plexiform, and the GCLs (249, 250, 251, 252). The lack of 7KChol metabolites in photodamaged retinas (at 48 h) may be due to the relatively rapid rate of mitochondrial and extramitochondrial generation of 7KChol compared with the rate of its mitochondrial metabolism by CYP27A1, consistent with the relatively slow retinal sterol turnover rate, and the rate of cholesterol accumulation in the CYP27A1 knockout mouse (204, 249, 250, 253). Elevated 7KChol levels have been observed in the RPE-choroid of rats with laser-induced choroidal neovascularization (CNV); the CNV was dramatically prevented by pretreatment with sterculic acid (197). In a knockout mouse model of hemochromatosis (a recessive human “iron-overload” disease), cholesterol and oxysterol accumulation has been attributed to decreased ABCA1 expression in the retina (129). The effects of in vivo administration of iron chelators on retinal oxysterol generation upon photodamage remain to be investigated.

The effects of oxysterols on retinal cells have been widely investigated in vitro using immortalized retina-derived cell lines and primary retinal cell. Although such treatments model the effects of an acute increase in membrane oxysterol levels, the approach is hindered by two significant drawbacks. First, the concentrations of oxysterols used to treat cells in culture may not faithfully mimic those generated under conditions of oxidative stress in the in vivo setting (see below). Second, the approach does not accurately model the effects of cellular enzymatic and nonenzymatic generation of oxysterols. A more physiologically relevant in vitro approach involves OxLDL treatment of cells, relying on the endocytic uptake of esterified oxysterols.

Cell death induced by 7KChol in R28 cells (an immortalized retinal progenitor cell line) was due to the activation of caspases 3, 8, and 12 (254, 255). Treatment of 661W cells (an immortalized mouse cone photoreceptor–derived cell line), rMC1 cells (an immortalized rat Müller glia–derived cell line), and primary monkey RPE cells with 7KChol demonstrated that 661W and rMC1 cell lines are significantly more sensitive to 7KChol cytotoxicity than are primary RPE cells (256). These in vitro assays suggest differential sensitivities of retinal cell types to 7KChol. (The effects of other highly cytotoxic, 7DHC-derived oxysterols on these retinal cell lines will be discussed later; see below.) OxLDL treatment was shown to induce PARP1-mediated cell death, Bax-mediated mitochondrial membrane permeabilization, and increased cellular ROS levels in cultured MIO-M1 human Müller glial cells (257). The increase in cellular ROS levels was accompanied by upregulation of NOX4 (a constitutively active member of the NADPH oxidase family of enzymes) and was reduced significantly by treatment with N-acetyl-cysteine (257).

Age-dependent accumulation of 7KChol in murine and monkey ocular tissues has been observed predominantly in Bruch's membrane and choroid and induces VEGF formation through nuclear factor kappa-B–mediated pathway (196, 210, 258). The 7KChol in Bruch's membrane serves as a chemoattractant, promoting microglial/macrophage migration, infiltration, and activation (198, 204, 258). Retinal microglias undergo cell death at the same dose range of 7KChol as other retinal cell types (256, 258). 7KChol induces lipid droplet accumulation in microglia, as well as their polarization to the pro-inflammatory M1 state (258). *In vitro* studies suggest that CD36-mediated 7KChol and OxLDL uptake induces inflammasome formation in endothelial cells and in retinal pericytes, the cellular components of the inner blood-retinal barrier (258, 259). LOX1 OxLDL receptor is expressed in endothelial cells and is involved in leukocyte-vascular interaction in various retinal inflammatory conditions (260).

We have thus far limited our discussion to the effects of three common oxysterols, that is, 24S-OH-Chol, 25S-OH-Chol, and 7KChol, on the RPE and the neural retina, or cells derived therefrom. Investigation of the effects of oxysterols and OxLDL in the neural retina is challenging because of the technical limitation of relevant animal models. Very few in vivo models exhibit chronic elevation in tissue and serum OxLDL, lipid hydroperoxide, and oxysterol levels. Exposure of the RPE and retina to lipid hydroperoxides by subretinal injection leads to accumulation of lipid droplets in the RPE, as well as CNV (261). Acute approaches, such as intravitreal injection of free 7KChol, result in direct incorporation of this cytotoxic oxysterol into all retinal cell types, which leads to panretinal degeneration and rapid retinal necrosis (within 1 week after injection) (262). Intravitreal injection of 7KChol also has been shown to cause lipid droplet accumulation, inflammation, and phagocytic defects in the RPE (263). Rapid increase in cellular 7KChol levels may overwhelm the rates of CYP27A1-mediated hydroxylation of 7KChol and the retinal cholesterol efflux rate, thereby leading to downstream cytotoxic effects (264). The protective role of CYP27A1-dependent metabolism of cytotoxic oxysterols is relevant in animal models where elevated retinal oxysterol levels have been demonstrated. Achieving *chronic* elevation of serum OxLDL levels, however, for example, by tail vein injection, may be difficult to achieve because of the rapid metabolism of serum OxLDL (265). Intravenous injection of mice with OxLDL only leads to transient increases (for less than *ca*. 15 min) in serum OxLDL levels, with the majority of the OxLDL ending up in the liver (265). Therefore, such approaches are of limited, if any, utility for studying the chronic effects of oxysterols or OxLDL on the neural retinal and the RPE in vivo.

### Lessons learned from animal models with pharmacological disruption of cholesterol synthesis

To date, only few animal models exhibit altered cholesterol homeostasis with chronic elevation of oxysterol and OxLDL levels. Serum OxLDL levels (specifically as involves 7KChol) are elevated in *Ldlr*^−/−^ and *ApoE*^−/−^ mice, which offer tractable animal models for studying atherosclerosis (266, 267). Elevated intraocular pressure (modeling glaucoma) induces increased retinal CYP46A1 activity, with resultant increase in 24-OH-Chol levels (268). However, none of these models exhibits chronic elevation of oxysterol levels, as is observed in diseases such as SLOS. The AY9944-induced rat model of SLOS has provided some key insights into the role of oxysterols (and possibly, by inference, OxLDL) in retinal degeneration (76). A unique feature of 7DHC, which accumulates in all bodily tissues and fluids of human patients with SLOS and in animal models with SLOS, is that it is the most readily oxidizable organic molecule known (75). 7DHC undergoes enzymatic and nonenzymatic (free radical–induced) oxidation, at rates 200-fold faster than cholesterol, and even seven-fold faster than DHA oxidation (75). This explains the formation and buildup of lipid peroxides and 7DHC-derived oxysterols in various tissue, including the retina, brain, liver, and blood, the rat model with SLOS, and patient samples (69, 262, 269). The observed retinal degeneration in the rat model with SLOS has been ascribed to two significant contributors: decreased cellular cholesterol content and increased 7DHC-derived, cytotoxic oxysterol levels. The cytotoxicity of 7DHC-derived oxysterols has been demonstrated in vitro using Neuro2a cell line, the 661W photoreceptor-derived cell line, the rMC-1 glial cell line, and in primary monkey RPE cells (256, 270). The lipidomic signatures of the SLOS rat retina includes elevated levels of 7DHC-derived oxysterols (which are minimal in age-matched controls), such as 7KChol, 4α-hydroxy-cholesta-5,7-dien-3β-ol, 4β-hydroxy-cholesta-5,7-dien-3β-ol, 24-hydroxy- cholesta-5,7-dien-3β-ol, and 3β,5α-dihydroxycholest-7-en-6-one. In fact, the 7KChol level in the retina of AY9944-treated rats is more than 50-fold higher than in age-matched controls (262, 269). The severity of the retinal degeneration observed in the SLOS rat model (at PN 5 weeks) was exacerbated by exposure to intense, constant light (1,700 lux, 24 h, at 480 nm), as compared with control rats subjected to the same conditions (271); however, the severity of the retinal degeneration was reduced by systemic administration of hydroxyl radical scavenger (dimethylthiourea), in agreement with previous studies suggesting protective effect upon light damage regimen (271, 272, 273). In addition, light damage also resulted in the marked elevation of lipid hydroperoxides in the retina AY9944-treated rats, compared with controls (274). Collectively, these observations strongly suggest a role for nonenzymatic oxidation of 7DHC in SLOS retinopathy. Hence, if one could block or substantially reduce the formation and accumulation of those oxysterols, the retinal degeneration might be avoided or the severity markedly reduced. This hypothesis was directly tested by evaluating the protective role of antioxidant supplementation, in addition to a high-cholesterol diet, in the AY9944-induced SLOS rat model. In an earlier study, a high-cholesterol diet (2%, by wt.) alone offered partial rescue of retinal degeneration; the severity was reduced but not fully prevented (79). On the other hand, supplementation of that same high-cholesterol diet with an antioxidant mixture (vitamins C and E, plus selenium) provided full protection against the retinal degeneration, and marked reduction in 7DHC-derived oxysterol levels in the retina and other tissues (275). The efficacy of antioxidant treatment is due to two important factors. First, although antioxidant supplementation did not fully inhibit oxysterol formation, it led to a significant decrease in the levels of 4β-hydroxy-cholesta-5,7-dien-3β-ol and 7KChol, compared with the diet supplemented with cholesterol alone. Hence, antioxidant-mediated protection in the SLOS model may be due to decreases in specific, cytotoxic oxysterol species. Second, the antioxidants used in the study function primarily through scavenging of a spectrum of cellular ROS species, thereby possibly also more broadly inhibiting lipid and protein peroxidation (not directly tested in the study), which have deleterious effects on the retina (discussed above). These observations provide compelling evidence for the key role for oxysterols in the observed retinal pathology (178, 276). Adapting similar pharmacological strategies, such as iron chelators or NADPH-oxidase inhibitors, in the SLOS model may further point to the contribution of cellular ROS, lipid peroxidation, protein peroxidation, and oxysterol formation in SLOS retinopathy. The retinal degeneration induced by distal inhibition of the mevalonate pathway (as observed in SLOS) and the rescue observed in the SLOS animal model upon cholesterol-antioxidant supplementation broadly demonstrate the importance of retinal cholesterol homeostasis to, and the deleterious effects of oxysterol formation on, the structure and function of the retina. It is worth noting that the rat model with SLOS develops a profound retinopathy starting at about 4–6 PN weeks of age, which is decidedly different from the slow, age-related onset (on the order of several months to a year) of retinal pathology in rodent models where cholesterol efflux has been disrupted (e.g., in ABC transporter/CYP enzyme knockout mouse models).

Here, it also should be noted that not all examples of disrupted cholesterol synthesis necessarily result in pathology, and also that certain animal models of human diseases may not faithfully recapitulate the human phenotype. An example of this is desmosterolosis, a severe, typically lethal, rare human genetic disorder caused by mutations in the gene encoding DHCR24 (OMIM #602398; EC 1.3.1.72) (277, 278), the enzyme that catalyzes the penultimate step in the mevalonate pathway whereby desmosterol (cholesta-5,24,-dien-3β–ol) is converted to cholesterol. A model of desmosterolosis was generated by treating rats with U18666A (3β-[2-(diethylamino)ethoxy]androst-5-en-17-one hydrochloride) (279, 280, 281), which has dual effects on both 2,3-oxidosqualene cyclase (EC 5.4.99.7) and (more prominently) DHCR24 (282, 283). Subsequently, this model was used to examine the effects of inhibiting DHCR24 on retinal structure and function (281, 284). Contrary to expectations, although rats treated with U18666A uniformly developed cataracts and had markedly elevated levels of desmosterol and reduced levels cholesterol in their serum and retinas (and, notably, in their rod OS membranes) compared with controls, the structure and function of the retina were remarkably normal (279, 284). Many years after the initial studies with U18666A were performed, an additional effect of the drug was discovered, that is, the allosteric inhibition of NPC-I (111, 281, 284, 285, 286). (See the prior discussion, above, regarding NPC-I–related retinopathy.)

## Technical advancements in studying cellular cholesterol and oxysterol distribution

Early investigations of tissues, including the retina, utilized freeze-fracture electron microscopic analysis of filipin-induced “pit” formation in cell membranes to determine the distribution of spatially distinct, cholesterol-rich membrane domains (subsequently thought to equate to “lipid rafts”) (287, 288, 289). In addition, the ability of digitonin to form one-to-one molecular complexes with cholesterol, followed by treatment with electron-dense contrast reagents, was leveraged to enable observation of sterol distribution by electron microscopy (290, 291). Filipin is also an endogenously fluorescent molecule (λ_exc_ = 360 nm, λ_em =_ 480 nm) (292), which has prompted its use as a probe to study cellular cholesterol distribution using fluorescence microscopy, although its utility is somewhat limited by its susceptibility to rapid photobleaching. Alternatively, fluorescent analogs of cholesterol, such as cholestatrienol or BODIPY-derivatized cholesterol, can be utilized to study cholesterol trafficking and distribution but require exogenous addition of the fluorescent analog. Similarly, fluor-tagged cholera toxin-B, which binds to GM1 (a ganglioside enriched, along with cholesterol, in lipid rafts) may be used to analyze lipid raft distribution (292). However, these strategies cannot be used for live-cell imaging because of the cytotoxic effects of these reagents. Hence, the information obtained from the more classical approaches mentioned above is limited to spatiotemporal snapshots of cholesterol distribution in cells and tissues at a given point in time, rather than affording dynamic monitoring of cholesterol trafficking and distribution in real time.

Perfringolysin O (PFO) is a membrane pore-forming bacterial Ɵ-toxin that binds to membrane cholesterol through its carboxy terminal D4 domain (293). Biotinylated PFO, in conjunction with streptavidin-gold, has been used to monitor cellular cholesterol distribution by electron microscopy (294). Fluor-tagged PFO D4 peptide also allows for monitoring of cholesterol distribution on the exofacial leaflet of cell membranes (295). Recently engineered chimeric D4-based probe (mutant D4 domain of PFO, D4H, tagged with mCherry) is available as expressible, noncytotoxic probe, which recognizes cholesterol on the cytosolic face of the membrane (295, 296, 297). Sterol distribution in cells also may be investigated using biorthogonal analogs of cholesterol, that is, analogs which can be integrated into a biological system without altering its natural biology, and allows monitoring of esterified and free cholesterol without cholesteryl esterase treatment, unlike filipin or mCherry-D4H. An alkyne analog of cholesterol, C19-alkyne cholesterol (or simply “eCholesterol”), is amenable to high-resolution microscopy imaging upon derivatization with a fluorescent azide using copper(I)-catalyzed alkyne-azide cycloaddition (298). Similar biorthogonal oxysterols, such as 25-OH-[C19-alkyne]-cholesterol (or simply, 25-OH-eCholesterol), have been developed, allowing for monitoring cellular distribution of oxysterols of interest (298, 299). Adaptation of such engineered probes or “click chemistry” in vitro in primary RPE and retinal neurons provides insights into the role of cholesterol in specialized cellular processes, such as RPE phagocytosis and neuronal synaptogenesis.

## Concluding remarks

To date, the fundamental processes involved in establishing and maintaining cholesterol homeostasis in the vertebrate retina appear to be fairly well understood, particularly under normal physiological conditions (see Fig. 3). However, there are a number of basic questions that still remain to be answered. Although studies using mice have suggested that at least 70% of the total cholesterol pool in the neural retina arises from endogenous de novo synthesis in the retina, it is not clear if that is generalizable to other animal species, including humans (60). Transcriptomic, RNASeq, in silico, and immunohistochemical studies of mevalonate pathway enzymes indicate that they predominantly are expressed in the RPE and *inner* retinal cells, rather than in the rod and cone photoreceptors, which would suggest that the photoreceptors cannot provide sufficient amounts of their own cholesterol via de novo synthesis to meet the demands of the cell even for new OS membrane biogenesis, let alone any other of the cell's myriad requirements for cholesterol (81, 82, 86, 104). Of the possible extracellular sources of cholesterol available to the photoreceptors, per se, it's still not clear what percentage comes from blood-borne lipoproteins, versus de novo synthesis by and secretion from RPE or Müller glial cells. OS disk membranes, which contain cholesterol, are assembled at the base of the OS, whereas an equivalent amount of “old” disk membranes is shed daily from the distal tips of the photoreceptors and then phagocytized and degraded by the RPE. Is some portion of the cholesterol that comprises those degraded disk membranes then recycled back to the photoreceptor cells? If so, what is the mechanism by which that occurs? What are the individual contributions of each neuronal cell type to the total de novo–synthesized pool of cholesterol found in the retina? Do those contributions vary depending upon physiological conditions, and, if so, how is that regulated? What is the turnover rate of de novo–synthesized cholesterol in the various cells types in the retina, and does this vary as a function of cell type? Although cholesterol appears to be the biologically preferred sterol in the vertebrate retina, why is it that some other sterols (e.g., desmosterol) appear to be similarly accommodated, whereas others (e.g., 7DHC) are not? Why are photoreceptor cells substantially more sensitive to the cytotoxic effects of certain oxysterols than other cells in the retina (e.g., RPE cells, Müller glia)? What determines the threshold concentration of various oxysterols, above which retinal cells degenerate and die? These and many other questions remain to be answered. With the advent of new tools, reagents, and techniques available now, which heretofore were either not developed or not validated, further research will address and answer these questions, thus filling in the remaining knowledge gaps concerning cholesterol homeostasis in the retina. As a prior publication pertinent to the topic of cholesterol homeostasis in the retina proclaimed, “the best is yet to come” (9).

## Conflicts of interest

The authors declare that they have no conflicts of interest with the contents of this article.

## References

[1] Bloch K. (1989). Sterol structure and function. Steroids.

[2] Luo J., Yang H., Song B.L. (2020). Mechanisms and regulation of cholesterol homeostasis. Nat. Rev. Mol. Cell Biol.

[3] Bjorkhem I., Meaney S. (2004). Brain cholesterol: long secret life behind a barrier. Arterioscler. Thromb. Vasc. Biol..

[4] Olkkonen V.M., Beaslas O., Nissila E. (2012). Oxysterols and their cellular effectors. Biomolecules.

[5] Griffiths W.J., Wang Y. (2019). Oxysterol research: a brief review. Biochem. Soc. Trans..

[6] Bjorkhem I., Cedazo-Minguez A., Leoni V., Meaney S. (2009). Oxysterols and neurodegenerative diseases. Mol. Aspects Med..

[7] Massey J.B., Pownall H.J. (2006). Structures of biologically active oxysterols determine their differential effects on phospholipid membranes. Biochemistry.

[8] Fliesler S.J., Bretillon L. (2010). The ins and outs of cholesterol in the vertebrate retina. J. Lipid Res..

[9] Pikuleva I.A., Curcio C.A. (2014). Cholesterol in the retina: the best is yet to come. Prog. Retin. Eye Res..

[10] Wolkow N., Song D., Song Y., Chu S., Hadziahmetovic M., Lee J.C. (2012). Ferroxidase hephaestin's cell-autonomous role in the retinal pigment epithelium. Am. J. Pathol..

[11] Arshavsky V.Y., Wensel T.G. (2013). Timing is everything: GTPase regulation in phototransduction. Invest. Ophthalmol. Vis. Sci..

[12] Fliesler S.J., Anderson R.E. (1983). Chemistry and metabolism of lipids in the vertebrate retina. Prog. Lipid Res..

[13] Molday R.S., Moritz O.L. (2015). Photoreceptors at a glance. J. Cell Sci.

[14] Young R.W. (1976). Visual cells and the concept of renewal. Invest. Ophthalmol. Vis. Sci..

[15] Chua N.K., Coates H.W., Brown A.J. (2020). Squalene monooxygenase: a journey to the heart of cholesterol synthesis. Prog. Lipid Res..

[16] Miziorko H.M. (2011). Enzymes of the mevalonate pathway of isoprenoid biosynthesis. Arch. Biochem. Biophys..

[17] Schroepfer G.J. (1981). Sterol biosynthesis. Annu. Rev. Biochem..

[18] Schroepfer G.J. (1982). Sterol biosynthesis. Annu. Rev. Biochem..

[19] Porter F.D., Herman G.E. (2011). Malformation syndromes caused by disorders of cholesterol synthesis. J. Lipid Res..

[20] Platt F.M., Wassif C., Colaco A., Dardis A., Lloyd-Evans E., Bembi B. (2014). Disorders of cholesterol metabolism and their unanticipated convergent mechanisms of disease. Annu. Rev. Genomics Hum. Genet..

[21] Siri-Tarino P.W., Krauss R.M. (2016). The early years of lipoprotein research: from discovery to clinical application. J. Lipid Res..

[22] Herz J., Bock H.H. (2002). Lipoprotein receptors in the nervous system. Annu. Rev. Biochem..

[23] Subramanian K., Balch W.E. (2008). NPC1/NPC2 function as a tag team duo to mobilize cholesterol. Proc. Natl. Acad. Sci. U.S.A..

[24] Linton K.J., Higgins C.F. (2007). Structure and function of ABC transporters: the ATP switch provides flexible control. Pflugers Arch..

[25] Oram J.F., Vaughan A.M. (2006). ATP-Binding cassette cholesterol transporters and cardiovascular disease. Circ. Res..

[26] Phillips M.C. (2018). Is ABCA1 a lipid transfer protein?. J. Lipid Res..

[27] Phillips M.C. (2014). Molecular mechanisms of cellular cholesterol efflux. J. Biol. Chem..

[28] Tang C., Oram J.F. (2009). The cell cholesterol exporter ABCA1 as a protector from cardiovascular disease and diabetes. Biochim. Biophys. Acta.

[29] Rebeck G.W. (2004). Cholesterol efflux as a critical component of Alzheimer's disease pathogenesis. J. Mol. Neurosci..

[30] Fredrickson D.S. (1964). The inheritance of high density lipoprotein deficiency (Tangier disease). J. Clin. Invest..

[31] Betzler B.K., Rim T.H., Sabanayagam C., Cheung C.M.G., Cheng C.Y. (2020). High-density lipoprotein cholesterol in age-related ocular diseases. Biomolecules.

[32] Nakamura K., Kennedy M.A., Baldan A., Bojanic D.D., Lyons K., Edwards P.A. (2004). Expression and regulation of multiple murine ATP-binding cassette transporter G1 mRNAs/isoforms that stimulate cellular cholesterol efflux to high density lipoprotein. J. Biol. Chem..

[33] Lehmann J.M., Kliewer S.A., Moore L.B., Smith-Oliver T.A., Oliver B.B., Su J.L. (1997). Activation of the nuclear receptor LXR by oxysterols defines a new hormone response pathway. J. Biol. Chem..

[34] Escher G., Krozowski Z., Croft K.D., Sviridov D. (2003). Expression of sterol 27-hydroxylase (CYP27A1) enhances cholesterol efflux. J. Biol. Chem..

[35] Theofilopoulos S., Griffiths W.J., Crick P.J., Yang S., Meljon A., Ogundare M. (2014). Cholestenoic acids regulate motor neuron survival via liver X receptors. J. Clin. Invest..

[36] Endo-Umeda K., Yasuda K., Sugita K., Honda A., Ohta M., Ishikawa M. (2014). 7-Dehydrocholesterol metabolites produced by sterol 27-hydroxylase (CYP27A1) modulate liver X receptor activity. J. Steroid Biochem. Mol. Biol..

[37] Javitt N.B. (2002). 25R,26-Hydroxycholesterol revisited: synthesis, metabolism, and biologic roles. J. Lipid Res..

[38] Griffiths W.J., Crick P.J., Meljon A., Theofilopoulos S., Abdel-Khalik J., Yutuc E. (2019). Additional pathways of sterol metabolism: evidence from analysis of Cyp27a1-/- mouse brain and plasma. Biochim. Biophys. Acta Mol. Cell Biol. Lipids.

[39] Salen G., Steiner R.D. (2017). Epidemiology, diagnosis, and treatment of cerebrotendinous xanthomatosis (CTX). J. Inherit. Metab. Dis..

[40] Nie S., Chen G., Cao X., Zhang Y. (2014). Cerebrotendinous xanthomatosis: a comprehensive review of pathogenesis, clinical manifestations, diagnosis, and management. Orphanet J. Rare Dis..

[41] Bjorkhem I., Diczfalusy U., Lovgren-Sandblom A., Starck L., Jonsson M., Tallman K. (2014). On the formation of 7-ketocholesterol from 7-dehydrocholesterol in patients with CTX and SLO. J. Lipid Res..

[42] Shinkyo R., Xu L., Tallman K.A., Cheng Q., Porter N.A., Guengerich F.P. (2011). Conversion of 7-dehydrocholesterol to 7-ketocholesterol is catalyzed by human cytochrome P450 7A1 and occurs by direct oxidation without an epoxide intermediate. J. Biol. Chem..

[43] Pikuleva I.A., Babiker A., Waterman M.R., Bjorkhem I. (1998). Activities of recombinant human cytochrome P450c27 (CYP27) which produce intermediates of alternative bile acid biosynthetic pathways. J. Biol. Chem..

[44] Marengo B., Bellora F., Ricciarelli R., De Ciucis C., Furfaro A., Leardi R. (2016). Oxysterol mixture and, in particular, 27-hydroxycholesterol drive M2 polarization of human macrophages. Biofactors.

[45] Anderson A., Campo A., Fulton E., Corwin A., Jerome W.G., O'Connor M.S. (2020). 7-Ketocholesterol in disease and aging. Redox Biol..

[46] Fliesler S.J., Schroepfer G.J. (1983). Metabolism of mevalonic acid in cell-free homogenates of bovine retinas. Formation of novel isoprenoid acids. J. Biol. Chem..

[47] Fliesler S.J., Schroepfer G.J. (1986). In vitro metabolism of mevalonic acid in the bovine retina. J. Neurochem..

[48] Fliesler S.J., Florman R., Rapp L.M., Pittler S.J., Keller R.K. (1993). In vivo biosynthesis of cholesterol in the rat retina. FEBS Lett..

[49] Pittler S.J., Fliesler S.J., Rapp L.M. (1992). Novel morphological changes in rat retina induced by intravitreal injection of lovastatin. Exp. Eye Res..

[50] Pittler S.J., Fliesler S.J., Fisher P.L., Keller P.K., Rapp L.M. (1995). In vivo requirement of protein prenylation for maintenance of retinal cytoarchitecture and photoreceptor structure. J. Cell Biol.

[51] Fliesler S.J., Keller R.K. (1995). Metabolism of [3H]farnesol to cholesterol and cholesterogenic intermediates in the living rat eye. Biochem. Biophys. Res. Commun..

[52] Laufs U., Liao J.K. (2003). Isoprenoid metabolism and the pleiotropic effects of statins. Curr. Atheroscler. Rep..

[53] Dietschy J.M., McGarry J.D. (1974). Limitations of acetate as a substrate for measuring cholesterol synthesis in liver. J. Biol. Chem..

[54] Previs S.F., Herath K., Nawrocki A.R., Rodriguez C.G., Slipetz D., Singh S.B. (2018). Using [(2)H]water to quantify the contribution of de novo palmitate synthesis in plasma: enabling back-to-back studies. Am. J. Physiol. Endocrinol. Metab..

[55] Roosing S., Collin R.W., den Hollander A.I., Cremers F.P., Siemiatkowska A.M. (2014). Prenylation defects in inherited retinal diseases. J. Med. Genet..

[56] Dietschy J.M., Spady D.K. (1984). Measurement of rates of cholesterol synthesis using tritiated water. J. Lipid Res..

[57] Jones P.J., Leitch C.A., Li Z.C., Connor W.E. (1993). Human cholesterol synthesis measurement using deuterated water. Theoretical and procedural considerations. Arterioscler. Thromb..

[58] Lee W.N., Bassilian S., Ajie H.O., Schoeller D.A., Edmond J., Bergner E.A. (1994). In vivo measurement of fatty acids and cholesterol synthesis using D2O and mass isotopomer analysis. Am. J. Physiol..

[59] Castro-Perez J., Previs S.F., McLaren D.G., Shah V., Herath K., Bhat G. (2011). In vivo D2O labeling to quantify static and dynamic changes in cholesterol and cholesterol esters by high resolution LC/MS. J. Lipid Res..

[60] Lin J.B., Mast N., Bederman I.R., Li Y., Brunengraber H., Bjorkhem I. (2016). Cholesterol in mouse retina originates primarily from in situ de novo biosynthesis. J. Lipid Res..

[61] Mast N., Bederman I.R., Pikuleva I.A. (2018). Retinal cholesterol content is reduced in Simvastatin-treated mice due to inhibited local biosynthesis albeit increased uptake of serum cholesterol. Drug Metab. Dispos.

[62] Zheng W., Mast N., Saadane A., Pikuleva I.A. (2015). Pathways of cholesterol homeostasis in mouse retina responsive to dietary and pharmacologic treatments. J. Lipid Res..

[63] Keller R.K., Fliesler S.J., Nellis S.W. (1988). Isoprenoid biosynthesis in the retina. Quantitation of the sterol and dolichol biosynthetic pathways. J. Biol. Chem..

[64] Crabtree B., Gordon M.J., Christie S.L. (1990). Measurement of the rates of acetyl-CoA hydrolysis and synthesis from acetate in rat hepatocytes and the role of these fluxes in substrate cycling. Biochem. J..

[65] Fliesler S.J., Florman R., Keller R.K. (1995). Isoprenoid lipid metabolism in the retina: dynamics of squalene and cholesterol incorporation and turnover in frog rod outer segment membranes. Exp. Eye Res..

[66] Mauch D.H., Nagler K., Schumacher S., Goritz C., Muller E.C., Otto A. (2001). CNS synaptogenesis promoted by glia-derived cholesterol. Science.

[67] Barres B.A., Smith S.J. (2001). Neurobiology. Cholesterol--making or breaking the synapse. Science.

[68] Porter F.D. (2008). Smith-Lemli-Opitz syndrome: pathogenesis, diagnosis and management. Eur. J. Hum. Genet..

[69] Yu H., Patel S.B. (2005). Recent insights into the Smith-Lemli-Opitz syndrome. Clin. Genet..

[70] Garry D., Hansen R.M., Moskowitz A., Elias E.R., Irons M., Fulton A.B. (2010). Cone ERG responses in patients with Smith-Lemli-Opitz Syndrome (SLOS). Doc. Ophthalmol..

[71] Elias E.R., Hansen R.M., Irons M., Quinn N.B., Fulton A.B. (2003). Rod photoreceptor responses in children with Smith-Lemli-Opitz syndrome. Arch. Ophthalmol..

[72] Xu L., Korade Z., Porter N.A. (2010). Oxysterols from free radical chain oxidation of 7-dehydrocholesterol: product and mechanistic studies. J. Am. Chem. Soc..

[73] Yin H., Xu L., Porter N.A. (2011). Free radical lipid peroxidation: mechanisms and analysis. Chem. Rev..

[74] Korade Z., Xu L., Shelton R., Porter N.A. (2010). Biological activities of 7-dehydrocholesterol-derived oxysterols: implications for Smith-Lemli-Opitz syndrome. J. Lipid Res..

[75] Xu L., Davis T.A., Porter N.A. (2009). Rate constants for peroxidation of polyunsaturated fatty acids and sterols in solution and in liposomes. J. Am. Chem. Soc..

[76] Fliesler S.J., Peachey N.S., Richards M.J., Nagel B.A., Vaughan D.K. (2004). Retinal degeneration in a rodent model of Smith-Lemli-Opitz syndrome: electrophysiologic, biochemical, and morphologic features. Arch. Ophthalmol..

[77] Ramachandra Rao S., Pfeffer B.A., Mas Gomez N., Skelton L.A., Keiko U., Sparrow J.R. (2018). Compromised phagosome maturation underlies RPE pathology in cell culture and whole animal models of Smith-Lemli-Opitz syndrome. Autophagy.

[78] Tu C., Li J., Jiang X., Sheflin L.G., Pfeffer B.A., Behringer M. (2013). Ion-current-based proteomic profiling of the retina in a rat model of Smith-Lemli-Opitz syndrome. Mol. Cell Proteomics.

[79] Fliesler S.J., Vaughan D.K., Jenewein E.C., Richards M.J., Nagel B.A., Peachey N.S. (2007). Partial rescue of retinal function and sterol steady-state in a rat model of Smith-Lemli-Opitz syndrome. Pediatr. Res..

[80] Tserentsoodol N., Sztein J., Campos M., Gordiyenko N.V., Fariss R.N., Lee J.W. (2006). Uptake of cholesterol by the retina occurs primarily via a low density lipoprotein receptor-mediated process. Mol. Vis..

[81] Zheng W., Reem R.E., Omarova S., Huang S., DiPatre P.L., Charvet C.D. (2012). Spatial distribution of the pathways of cholesterol homeostasis in human retina. PLoS One.

[82] Collin J., Zerti D., Queen R., Santos-Ferreira T., Bauer R., Coxhead J. (2019). CRX Expression in pluripotent stem cell-derived photoreceptors marks a transplantable subpopulation of early cones. Stem Cells.

[83] Glubrecht D.D., Kim J.H., Russell L., Bamforth J.S., Godbout R. (2009). Differential CRX and OTX2 expression in human retina and retinoblastoma. J. Neurochem..

[84] Prasov L., Glaser T. (2012). Pushing the envelope of retinal ganglion cell genesis: context dependent function of Math5 (Atoh7). Dev. Biol..

[85] Furukawa A., Koike C., Lippincott P., Cepko C.L., Furukawa T. (2002). The mouse Crx 5'-upstream transgene sequence directs cell-specific and developmentally regulated expression in retinal photoreceptor cells. J. Neurosci..

[86] Zekavat S.M., Lu J., Maugeais C., Mazer N.A. (2017). An in silico model of retinal cholesterol dynamics (RCD model): insights into the pathophysiology of dry AMD. J. Lipid Res..

[87] Young R.W., Bok D. (1969). Participation of the retinal pigment epithelium in the rod outer segment renewal process. J. Cell Biol.

[88] Klimova L., Lachova J., Machon O., Sedlacek R., Kozmik Z. (2013). Generation of mRx-Cre transgenic mouse line for efficient conditional gene deletion in early retinal progenitors. PLoS One.

[89] Amaratunga A., Abraham C.R., Edwards R.B., Sandell J.H., Schreiber B.M., Fine R.E. (1996). Apolipoprotein E is synthesized in the retina by Muller glial cells, secreted into the vitreous, and rapidly transported into the optic nerve by retinal ganglion cells. J. Biol. Chem..

[90] Roesch K., Jadhav A.P., Trimarchi J.M., Stadler M.B., Roska B., Sun B.B. (2008). The transcriptome of retinal Muller glial cells. J. Comp. Neurol..

[91] Shanmugaratnam J., Berg E., Kimerer L., Johnson R.J., Amaratunga A., Schreiber B.M. (1997). Retinal Muller glia secrete apolipoproteins E and J which are efficiently assembled into lipoprotein particles. Brain Res. Mol. Brain Res..

[92] Fagan A.M., Holtzman D.M., Munson G., Mathur T., Schneider D., Chang L.K. (1999). Unique lipoproteins secreted by primary astrocytes from wild type, apoE (-/-), and human apoE transgenic mice. J. Biol. Chem..

[93] Saadane A., Petrov A., Mast N., El-Darzi N., Dao T., Alnemri A. (2018). Mechanisms that minimize retinal impact of apolipoprotein E absence. J. Lipid Res..

[94] Ueki Y., Ash J.D., Zhu M., Zheng L., Le Y.Z. (2009). Expression of Cre recombinase in retinal Muller cells. Vis. Res.

[95] Shen W., Fruttiger M., Zhu L., Chung S.H., Barnett N.L., Kirk J.K. (2012). Conditional Mullercell ablation causes independent neuronal and vascular pathologies in a novel transgenic model. J. Neurosci..

[96] Ong J.M., Zorapapel N.C., Rich K.A., Wagstaff R.E., Lambert R.W., Rosenberg S.E. (2001). Effects of cholesterol and apolipoprotein E on retinal abnormalities in ApoE-deficient mice. Invest. Ophthalmol. Vis. Sci..

[97] Kurumada S., Onishi A., Imai H., Ishii K., Kobayashi T., Sato S.B. (2007). Stage-specific association of apolipoprotein A-I and E in developing mouse retina. Invest. Ophthalmol. Vis. Sci..

[98] Lin J.P., Mironova Y.A., Shrager P., Giger R.J. (2017). LRP1 regulates peroxisome biogenesis and cholesterol homeostasis in oligodendrocytes and is required for proper CNS myelin development and repair. Elife.

[99] Landowski L.M., Pavez M., Brown L.S., Gasperini R., Taylor B.V., West A.K. (2016). Low-density lipoprotein receptor-related proteins in a novel mechanism of axon guidance and peripheral nerve regeneration. J. Biol. Chem..

[100] Hollborn M., Birkenmeier G., Saalbach A., Iandiev I., Reichenbach A., Wiedemann P. (2004). Expression of LRP1 in retinal pigment epithelial cells and its regulation by growth factors. Invest. Ophthalmol. Vis. Sci..

[101] Mao H., Lockyer P., Townley-Tilson W.H., Xie L., Pi X. (2016). LRP1 regulates retinal angiogenesis by inhibiting PARP-1 activity and endothelial cell proliferation. Arterioscler. Thromb. Vasc. Biol..

[102] Barcelona P.F., Ortiz S.G., Chiabrando G.A., Sanchez M.C. (2011). alpha2-Macroglobulin induces glial fibrillary acidic protein expression mediated by low-density lipoprotein receptor-related protein 1 in Muller cells. Invest. Ophthalmol. Vis. Sci..

[103] Biswas L., Zhou X., Dhillon B., Graham A., Shu X. (2017). Retinal pigment epithelium cholesterol efflux mediated by the 18 kDa translocator protein, TSPO, a potential target for treating age-related macular degeneration. Hum. Mol. Genet..

[104] Louer E.M.M., Yi G., Carmone C., Robben J., Stunnenberg H.G., den Hollander A.I. (2020). Genes involved in energy metabolism are differentially expressed during the day-night cycle in murine retinal pigment epithelium. Invest. Ophthalmol. Vis. Sci..

[105] Gordiyenko N., Campos M., Lee J.W., Fariss R.N., Sztein J., Rodriguez I.R. (2004). RPE cells internalize low-density lipoprotein (LDL) and oxidized LDL (oxLDL) in large quantities in vitro and in vivo. Invest. Ophthalmol. Vis. Sci..

[106] Yin L., Shi Y., Liu X., Zhang H., Gong Y., Gu Q. (2012). A rat model for studying the biological effects of circulating LDL in the choriocapillaris-BrM-RPE complex. Am. J. Pathol..

[107] Mishra S., Peterson K., Yin L., Berger A., Fan J., Wistow G. (2016). Accumulation of cholesterol and increased demand for zinc in serum-deprived RPE cells. Mol. Vis..

[108] Bretillon L., Acar N., Seeliger M.W., Santos M., Maire M.A., Juaneda P. (2008). ApoB100,LDLR-/- mice exhibit reduced electroretinographic response and cholesteryl esters deposits in the retina. Invest. Ophthalmol. Vis. Sci..

[109] Lakkaraju A., Finnemann S.C., Rodriguez-Boulan E. (2007). The lipofuscin fluorophore A2E perturbs cholesterol metabolism in retinal pigment epithelial cells. Proc. Natl. Acad. Sci. U.S.A..

[110] Ory D.S. (2004). The niemann-pick disease genes; regulators of cellular cholesterol homeostasis. Trends Cardiovasc. Med..

[111] Claudepierre T., Paques M., Simonutti M., Buard I., Sahel J., Maue R.A. (2010). Lack of Niemann-Pick type C1 induces age-related degeneration in the mouse retina. Mol. Cell Neurosci.

[112] Yan X., Ma L., Hovakimyan M., Lukas J., Wree A., Frank M. (2014). Defects in the retina of Niemann-pick type C 1 mutant mice. BMC Neurosci..

[113] Havla J., Moser M., Sztatecsny C., Lotz-Havla A.S., Maier E.M., Hizli B. (2020). Retinal axonal degeneration in Niemann-Pick type C disease. J. Neurol..

[114] Cohen J.L., Burfield J., Valdez-Gonzalez K., Samuels A., Stefanatos A.K., Yudkoff M. (2019). Early diagnosis of infantile-onset lysosomal acid lipase deficiency in the advent of available enzyme replacement therapy. Orphanet J. Rare Dis..

[115] Elner V.M. (2002). Retinal pigment epithelial acid lipase activity and lipoprotein receptors: effects of dietary omega-3 fatty acids. Trans. Am. Ophthalmol. Soc..

[116] Hayes K.C., Lindsey S., Stephan Z.F., Brecker D. (1989). Retinal pigment epithelium possesses both LDL and scavenger receptor activity. Invest. Ophthalmol. Vis. Sci..

[117] Duncan K.G., Bailey K.R., Kane J.P., Schwartz D.M. (2002). Human retinal pigment epithelial cells express scavenger receptors BI and BII. Biochem. Biophys. Res. Commun..

[118] Sato Y., Kondo Y., Sumi M., Takekuma Y., Sugawara M. (2013). Intracellular uptake mechanism of lutein in retinal pigment epithelial cells. J. Pharm. Pharm. Sci..

[119] Shen W.J., Azhar S., Kraemer F.B. (2018). SR-B1: a unique multifunctional receptor for cholesterol influx and efflux. Annu. Rev. Physiol..

[120] Provost A.C., Vede L., Bigot K., Keller N., Tailleux A., Jais J.P. (2009). Morphologic and electroretinographic phenotype of SR-BI knockout mice after a long-term atherogenic diet. Invest. Ophthalmol. Vis. Sci..

[121] Martin S., Parton R.G. (2005). Caveolin, cholesterol, and lipid bodies. Semin. Cell Dev. Biol.

[122] Olofsson S.O., Bostrom P., Andersson L., Rutberg M., Perman J., Boren J. (2009). Lipid droplets as dynamic organelles connecting storage and efflux of lipids. Biochim. Biophys. Acta.

[123] Rogers M.A., Liu J., Song B.L., Li B.L., Chang C.C., Chang T.Y. (2015). Acyl-CoA:cholesterol acyltransferases (ACATs/SOATs): enzymes with multiple sterols as substrates and as activators. J. Steroid Biochem. Mol. Biol..

[124] Li C.M., Presley J.B., Zhang X., Dashti N., Chung B.H., Medeiros N.E. (2005). Retina expresses microsomal triglyceride transfer protein: implications for age-related maculopathy. J. Lipid Res..

[125] Saadane A., Mast N., Dao T., Ahmad B., Pikuleva I.A. (2016). Retinal hypercholesterolemia triggers cholesterol accumulation and esterification in photoreceptor cells. J. Biol. Chem..

[126] Sakai N., Vaisman B.L., Koch C.A., Hoyt R.F., Meyn S.M., Talley G.D. (1997). Targeted disruption of the mouse lecithin:cholesterol acyltransferase (LCAT) gene. Generation of a new animal model for human LCAT deficiency. J. Biol. Chem..

[127] Bojanic D.D., Tarr P.T., Gale G.D., Smith D.J., Bok D., Chen B. (2010). Differential expression and function of ABCG1 and ABCG4 during development and aging. J. Lipid Res..

[128] Xu M., Zhou H., Tan K.C., Guo R., Shiu S.W., Wong Y. (2009). ABCG1 mediated oxidized LDL-derived oxysterol efflux from macrophages. Biochem. Biophys. Res. Commun..

[129] Ananth S., Gnana-Prakasam J.P., Bhutia Y.D., Veeranan-Karmegam R., Martin P.M., Smith S.B. (2014). Regulation of the cholesterol efflux transporters ABCA1 and ABCG1 in retina in hemochromatosis and by the endogenous siderophore 2,5-dihydroxybenzoic acid. Biochim. Biophys. Acta.

[130] Li S., Chen D., Sauve Y., McCandless J., Chen Y.J., Chen C.K. (2005). Rhodopsin-iCre transgenic mouse line for Cre-mediated rod-specific gene targeting. Genesis.

[131] Ban N., Lee T.J., Sene A., Dong Z., Santeford A., Lin J.B. (2018). Disrupted cholesterol metabolism promotes age-related photoreceptor neurodegeneration. J. Lipid Res..

[132] Choudhary M., Ismail E.N., Yao P.L., Tayyari F., Radu R.A., Nusinowitz S. (2020). LXRs regulate features of age-related macular degeneration and may be a potential therapeutic target. JCI Insight.

[133] Song X.Y., Wu W.F., Gabbi C., Dai Y.B., So M., Chaurasiya S.P. (2019). Retinal and optic nerve degeneration in liver X receptor beta knockout mice. Proc. Natl. Acad. Sci. U.S.A..

[134] Storti F., Klee K., Todorova V., Steiner R., Othman A., van der Velde-Visser S. (2019). Impaired ABCA1/ABCG1-mediated lipid efflux in the mouse retinal pigment epithelium (RPE) leads to retinal degeneration. Elife.

[135] Storti F., Raphael G., Griesser V., Klee K., Drawnel F., Willburger C. (2017). Regulated efflux of photoreceptor outer segment-derived cholesterol by human RPE cells. Exp. Eye Res..

[136] Simo R., Garcia-Ramirez M., Higuera M., Hernandez C. (2009). Apolipoprotein A1 is overexpressed in the retina of diabetic patients. Am. J. Ophthalmol..

[137] Li C.M., Chung B.H., Presley J.B., Malek G., Zhang X., Dashti N. (2005). Lipoprotein-like particles and cholesteryl esters in human Bruch's membrane: initial characterization. Invest. Ophthalmol. Vis. Sci..

[138] Lyssenko N.N., Haider N., Picataggi A., Cipollari E., Jiao W., Phillips M.C. (2018). Directional ABCA1-mediated cholesterol efflux and apoB-lipoprotein secretion in the retinal pigment epithelium. J. Lipid Res..

[139] Pilgrim M.G., Lengyel I., Lanzirotti A., Newville M., Fearn S., Emri E. (2017). Subretinal pigment epithelial deposition of drusen components including hydroxyapatite in a primary cell culture model. Invest. Ophthalmol. Vis. Sci..

[140] Pfeffer B.A., Philp N.J. (2014). Cell culture of retinal pigment epithelium: special issue. Exp. Eye Res..

[141] Ban N., Lee T.J., Sene A., Choudhary M., Lekwuwa M., Dong Z. (2018). Impaired monocyte cholesterol clearance initiates age-related retinal degeneration and vision loss. JCI Insight.

[142] Zhao L., Zabel M.K., Wang X., Ma W., Shah P., Fariss R.N. (2015). Microglial phagocytosis of living photoreceptors contributes to inherited retinal degeneration. EMBO Mol. Med..

[143] Levy O., Calippe B., Lavalette S., Hu S.J., Raoul W., Dominguez E. (2015). Apolipoprotein E promotes subretinal mononuclear phagocyte survival and chronic inflammation in age-related macular degeneration. EMBO Mol. Med..

[144] Ramachandra Rao S., Skelton L.A., Wu F., Onysk A., Spolnik G., Danikiewicz W. (2020). Retinal degeneration caused by rod-specific Dhdds ablation occurs without concomitant inhibition of protein N-glycosylation. iScience.

[145] Mast N., Reem R., Bederman I., Huang S., DiPatre P.L., Bjorkhem I. (2011). Cholestenoic Acid is an important elimination product of cholesterol in the retina: comparison of retinal cholesterol metabolism with that in the brain. Invest. Ophthalmol. Vis. Sci..

[146] Lee J.W., Fuda H., Javitt N.B., Strott C.A., Rodriguez I.R. (2006). Expression and localization of sterol 27-hydroxylase (CYP27A1) in monkey retina. Exp. Eye Res..

[147] Charvet C., Liao W.L., Heo G.Y., Laird J., Salomon R.G., Turko I.V. (2011). Isolevuglandins and mitochondrial enzymes in the retina: mass spectrometry detection of post-translational modification of sterol-metabolizing CYP27A1. J. Biol. Chem..

[148] Rupprecht R., Papadopoulos V., Rammes G., Baghai T.C., Fan J., Akula N. (2010). Translocator protein (18 kDa) (TSPO) as a therapeutic target for neurological and psychiatric disorders. Nat. Rev. Drug Discov..

[149] Costa B., Da Pozzo E., Martini C. (2020). 18-kDa translocator protein association complexes in the brain: from structure to function. Biochem. Pharmacol..

[150] Graham A. (2015). Mitochondrial regulation of macrophage cholesterol homeostasis. Free Radic. Biol. Med..

[151] Alamri A., Biswas L., Watson D.G., Shu X. (2019). Deletion of TSPO resulted in change of metabolomic profile in retinal pigment epithelial cells. Int. J. Mol. Sci..

[152] Nakano M., Kelly E.J., Wiek C., Hanenberg H., Rettie A.E. (2012). CYP4V2 in Bietti's crystalline dystrophy: ocular localization, metabolism of omega-3-polyunsaturated fatty acids, and functional deficit of the p.H331P variant. Mol. Pharmacol..

[153] Hata M., Ikeda H.O., Iwai S., Iida Y., Gotoh N., Asaka I. (2018). Reduction of lipid accumulation rescues Bietti's crystalline dystrophy phenotypes. Proc. Natl. Acad. Sci. U.S.A..

[154] Lutjohann D., Breuer O., Ahlborg G., Nennesmo I., Siden A., Diczfalusy U. (1996). Cholesterol homeostasis in human brain: evidence for an age-dependent flux of 24S-hydroxycholesterol from the brain into the circulation. Proc. Natl. Acad. Sci. U.S.A..

[155] Bjorkhem I., Lutjohann D., Breuer O., Sakinis A., Wennmalm A. (1997). Importance of a novel oxidative mechanism for elimination of brain cholesterol. Turnover of cholesterol and 24(S)-hydroxycholesterol in rat brain as measured with 18O2 techniques in vivo and in vitro. J. Biol. Chem..

[156] Russell D.W., Halford R.W., Ramirez D.M., Shah R., Kotti T. (2009). Cholesterol 24-hydroxylase: an enzyme of cholesterol turnover in the brain. Annu. Rev. Biochem..

[157] Bretillon L., Diczfalusy U., Bjorkhem I., Maire M.A., Martine L., Joffre C. (2007). Cholesterol-24S-hydroxylase (CYP46A1) is specifically expressed in neurons of the neural retina. Curr. Eye Res..

[158] Gao H., Pennesi M.E., Shah K., Qiao X., Hariprasad S.M., Mieler W.F. (2004). Intravitreal voriconazole: an electroretinographic and histopathologic study. Arch. Ophthalmol..

[159] Fourgeux C., Martine L., Acar N., Bron A.M., Creuzot-Garcher C.P., Bretillon L. (2014). In vivo consequences of cholesterol-24S-hydroxylase (CYP46A1) inhibition by voriconazole on cholesterol homeostasis and function in the rat retina. Biochem. Biophys. Res. Commun..

[160] Liao W.L., Heo G.Y., Dodder N.G., Reem R.E., Mast N., Huang S. (2011). Quantification of cholesterol-metabolizing P450s CYP27A1 and CYP46A1 in neural tissues reveals a lack of enzyme-product correlations in human retina but not human brain. J. Proteome Res..

[161] Zhang J., Akwa Y., el-Etr M., Baulieu E.E., Sjovall J. (1997). Metabolism of 27-, 25- and 24-hydroxycholesterol in rat glial cells and neurons. Biochem. J..

[162] Saadane A., Mast N., Trichonas G., Chakraborty D., Hammer S., Busik J.V. (2019). Retinal Vascular abnormalities and microglia activation in mice with deficiency in cytochrome P450 46A1-mediated cholesterol removal. Am. J. Pathol..

[163] Saadane A., Mast N., Charvet C.D., Omarova S., Zheng W., Huang S.S. (2014). Retinal and nonocular abnormalities in Cyp27a1(-/-)Cyp46a1(-/-) mice with dysfunctional metabolism of cholesterol. Am. J. Pathol..

[164] Schroepfer G.J. (2000). Oxysterols: modulators of cholesterol metabolism and other processes. Physiol. Rev..

[165] Brown A.J., Jessup W. (2009). Oxysterols: sources, cellular storage and metabolism, and new insights into their roles in cholesterol homeostasis. Mol. Aspects Med..

[166] Massey J.B. (2006). Membrane and protein interactions of oxysterols. Curr. Opin. Lipidol..

[167] Tanito M., Haniu H., Elliott M.H., Singh A.K., Matsumoto H., Anderson R.E. (2006). Identification of 4-hydroxynonenal-modified retinal proteins induced by photooxidative stress prior to retinal degeneration. Free Radic. Biol. Med..

[168] Barnaba C., Rodriguez-Estrada M.T., Lercker G., Garcia H.S., Medina-Meza I.G. (2016). Cholesterol photo-oxidation: a chemical reaction network for kinetic modeling. Steroids.

[169] Iuliano L. (2011). Pathways of cholesterol oxidation via non-enzymatic mechanisms. Chem. Phys. Lipids.

[170] Murphy R.C., Johnson K.M. (2008). Cholesterol, reactive oxygen species, and the formation of biologically active mediators. J. Biol. Chem..

[171] Carvalho J.F., Silva M.M., Moreira J.N., Simoes S., Sa E.M.M.L. (2011). Selective cytotoxicity of oxysterols through structural modulation on rings A and B. Synthesis, in vitro evaluation, and SAR. J. Med. Chem..

[172] O'Callaghan Y.C., Woods J.A., O'Brien N.M. (2001). Comparative study of the cytotoxicity and apoptosis-inducing potential of commonly occurring oxysterols. Cell Biol Toxicol.

[173] Braughler J.M., Duncan L.A., Chase R.L. (1986). The involvement of iron in lipid peroxidation. Importance of ferric to ferrous ratios in initiation. J. Biol. Chem..

[174] Latunde-Dada G.O. (2017). Ferroptosis: role of lipid peroxidation, iron and ferritinophagy. Biochim. Biophys. Acta Gen. Subj.

[175] He X., Hahn P., Iacovelli J., Wong R., King C., Bhisitkul R. (2007). Iron homeostasis and toxicity in retinal degeneration. Prog. Retin. Eye Res..

[176] Loh A., Hadziahmetovic M., Dunaief J.L. (2009). Iron homeostasis and eye disease. Biochim. Biophys. Acta.

[177] Filomenko R., Fourgeux C., Bretillon L., Gambert-Nicot S. (2015). Oxysterols: influence on plasma membrane rafts microdomains and development of ocular diseases. Steroids.

[178] Fliesler S.J., Xu L. (2018). Oxysterols and retinal degeneration in a rat model of Smith-Lemli-Opitz syndrome: implications for an improved therapeutic intervention. Molecules.

[179] Kuznetsova A.V., Kurinov A.M., Aleksandrova M.A. (2014). Cell models to study regulation of cell transformation in pathologies of retinal pigment epithelium. J. Ophthalmol..

[180] Fronk A.H., Vargis E. (2016). Methods for culturing retinal pigment epithelial cells: a review of current protocols and future recommendations. J. Tissue Eng..

[181] Ryeom S.W., Sparrow J.R., Silverstein R.L. (1996). CD36 participates in the phagocytosis of rod outer segments by retinal pigment epithelium. J. Cell Sci.

[182] Finnemann S.C., Silverstein R.L. (2001). Differential roles of CD36 and alphavbeta5 integrin in photoreceptor phagocytosis by the retinal pigment epithelium. J. Exp. Med..

[183] Ryeom S.W., Silverstein R.L., Scotto A., Sparrow J.R. (1996). Binding of anionic phospholipids to retinal pigment epithelium may be mediated by the scavenger receptor CD36. J. Biol. Chem..

[184] Sun M., Finnemann S.C., Febbraio M., Shan L., Annangudi S.P., Podrez E.A. (2006). Light-induced oxidation of photoreceptor outer segment phospholipids generates ligands for CD36-mediated phagocytosis by retinal pigment epithelium: a potential mechanism for modulating outer segment phagocytosis under oxidant stress conditions. J. Biol. Chem..

[185] Gnanaguru G., Choi A.R., Amarnani D., D'Amore P.A. (2016). Oxidized lipoprotein uptake through the CD36 receptor activates the NLRP3 inflammasome in human retinal pigment epithelial cells. Invest. Ophthalmol. Vis. Sci..

[186] Hoppe G., Marmorstein A.D., Pennock E.A., Hoff H.F. (2001). Oxidized low density lipoprotein-induced inhibition of processing of photoreceptor outer segments by RPE. Invest. Ophthalmol. Vis. Sci..

[187] Hoppe G., O'Neil J., Hoff H.F., Sears J. (2004). Accumulation of oxidized lipid-protein complexes alters phagosome maturation in retinal pigment epithelium. Cell Mol. Life Sci.

[188] Hoppe G., O'Neil J., Hoff H.F., Sears J. (2004). Products of lipid peroxidation induce missorting of the principal lysosomal protease in retinal pigment epithelium. Biochim. Biophys. Acta.

[189] Yu A.L., Lorenz R.L., Haritoglou C., Kampik A., Welge-Lussen U. (2009). Biological effects of native and oxidized low-density lipoproteins in cultured human retinal pigment epithelial cells. Exp. Eye Res..

[190] Yin L., Wu X., Gong Y., Shi Y., Qiu Y., Zhang H. (2011). OX-LDL up-regulates the vascular endothelial growth factor-to-pigment epithelium-derived factor ratio in human retinal pigment epithelial cells. Curr. Eye Res..

[191] Kim J.H., Lee S.J., Kim K.W., Yu Y.S., Kim J.H. (2012). Oxidized low density lipoprotein-induced senescence of retinal pigment epithelial cells is followed by outer blood-retinal barrier dysfunction. Int. J. Biochem. Cell Biol.

[192] Yating Q., Yuan Y., Wei Z., Qing G., Xingwei W., Qiu Q. (2015). Oxidized LDL induces apoptosis of human retinal pigment epithelium through activation of ERK-Bax/Bcl-2 signaling pathways. Curr. Eye Res..

[193] Ong J.M., Aoki A.M., Seigel G.M., Sacerio I., Castellon R., Nesburn A.B. (2003). Oxysterol-induced toxicity in R28 and ARPE-19 cells. Neurochem. Res..

[194] Dugas B., Charbonnier S., Baarine M., Ragot K., Delmas D., Menetrier F. (2010). Effects of oxysterols on cell viability, inflammatory cytokines, VEGF, and reactive oxygen species production on human retinal cells: cytoprotective effects and prevention of VEGF secretion by resveratrol. Eur. J. Nutr..

[195] Gramajo A.L., Zacharias L.C., Neekhra A., Luthra S., Atilano S.R., Chwa M. (2010). Mitochondrial DNA damage induced by 7-ketocholesterol in human retinal pigment epithelial cells in vitro. Invest. Ophthalmol. Vis. Sci..

[196] Moreira E.F., Larrayoz I.M., Lee J.W., Rodriguez I.R. (2009). 7-Ketocholesterol is present in lipid deposits in the primate retina: potential implication in the induction of VEGF and CNV formation. Invest. Ophthalmol. Vis. Sci..

[197] Huang J.D., Amaral J., Lee J.W., Larrayoz I.M., Rodriguez I.R. (2012). Sterculic acid antagonizes 7-ketocholesterol-mediated inflammation and inhibits choroidal neovascularization. Biochim. Biophys. Acta.

[198] Larrayoz I.M., Huang J.D., Lee J.W., Pascual I., Rodriguez I.R. (2010). 7-ketocholesterol-induced inflammation: involvement of multiple kinase signaling pathways via NFkappaB but independently of reactive oxygen species formation. Invest. Ophthalmol. Vis. Sci..

[199] Olivier E., Dutot M., Regazzetti A., Leguillier T., Dargere D., Auzeil N. (2016). P2X7-pannexin-1 and amyloid beta-induced oxysterol input in human retinal cell: role in age-related macular degeneration?. Biochimie.

[200] Joffre C., Leclere L., Buteau B., Martine L., Cabaret S., Malvitte L. (2007). Oxysterols induced inflammation and oxidation in primary porcine retinal pigment epithelial cells. Curr. Eye Res..

[201] Shi G., Chen S., Wandu W.S., Ogbeifun O., Nugent L.F., Maminishkis A. (2015). Inflammasomes Induced by 7-Ketocholesterol and Other Stimuli in RPE and in Bone Marrow-Derived Cells Differ Markedly in Their Production of IL-1beta and IL-18. Invest. Ophthalmol. Vis. Sci..

[202] Lee J.W., Huang J.D., Rodriguez I.R. (2015). Extra-hepatic metabolism of 7-ketocholesterol occurs by esterification to fatty acids via cPLA2alpha and SOAT1 followed by selective efflux to HDL. Biochim. Biophys. Acta.

[203] Catarino S., Bento C.F., Brito A., Murteira E., Fernandes A.F., Pereira P. (2012). Regulation of the expression of interleukin-8 induced by 25-hydroxycholesterol in retinal pigment epithelium cells. Acta Ophthalmol..

[204] Rodriguez I.R., Larrayoz I.M. (2010). Cholesterol oxidation in the retina: implications of 7KCh formation in chronic inflammation and age-related macular degeneration. J. Lipid Res..

[205] Curcio C.A. (2018). Antecedents of soft drusen, the specific deposits of age-related macular degeneration, in the biology of human macula. Invest. Ophthalmol. Vis. Sci..

[206] Wang L., Clark M.E., Crossman D.K., Kojima K., Messinger J.D., Mobley J.A. (2010). Abundant lipid and protein components of drusen. PLoS One.

[207] Rudolf M., Seckerdieck K., Grisanti S., Curcio C.A. (2014). Internal structure consistent with remodelling in very small drusen, revealed by filipin histochemistry for esterified cholesterol. Br. J. Ophthalmol..

[208] Curcio C.A. (2018). Soft drusen in age-related macular degeneration: biology and targeting via the oil spill strategies. Invest. Ophthalmol. Vis. Sci..

[209] Johnson L.V., Forest D.L., Banna C.D., Radeke C.M., Maloney M.A., Hu J. (2011). Cell culture model that mimics drusen formation and triggers complement activation associated with age-related macular degeneration. Proc. Natl. Acad. Sci. U.S.A..

[210] Rodriguez I.R., Clark M.E., Lee J.W., Curcio C.A. (2014). 7-ketocholesterol accumulates in ocular tissues as a consequence of aging and is present in high levels in drusen. Exp. Eye Res..

[211] Crabb J.W., Miyagi M., Gu X., Shadrach K., West K.A., Sakaguchi H. (2002). Drusen proteome analysis: an approach to the etiology of age-related macular degeneration. Proc. Natl. Acad. Sci. U.S.A..

[212] Crabb J.W. (2014). The proteomics of drusen. Cold Spring Harb. Perspect. Med..

[213] Javitt N.B., Javitt J.C. (2009). The retinal oxysterol pathway: a unifying hypothesis for the cause of age-related macular degeneration. Curr. Opin. Ophthalmol..

[214] Klein R., Lee K.E., Tsai M.Y., Cruickshanks K.J., Gangnon R.E., Klein B.E.K. (2019). Oxidized low-density lipoprotein and the incidence of age-related macular degeneration. Ophthalmology.

[215] Pariente A., Pelaez R., Perez-Sala A., Larrayoz I.M. (2019). Inflammatory and cell death mechanisms induced by 7-ketocholesterol in the retina. Implications for age-related macular degeneration. Exp. Eye Res..

[216] Sparrow J.R., Fishkin N., Zhou J., Cai B., Jang Y.P., Krane S. (2003). A2E, a byproduct of the visual cycle. Vis. Res.

[217] Toops K.A., Tan L.X., Jiang Z., Radu R.A., Lakkaraju A. (2015). Cholesterol-mediated activation of acid sphingomyelinase disrupts autophagy in the retinal pigment epithelium. Mol. Biol. Cell.

[218] Tan L.X., Toops K.A., Lakkaraju A. (2016). Protective responses to sublytic complement in the retinal pigment epithelium. Proc. Natl. Acad. Sci. U.S.A..

[219] Rai A., Pathak D., Thakur S., Singh S., Dubey A.K., Mallik R. (2016). Dynein clusters into lipid microdomains on phagosomes to drive rapid transport toward lysosomes. Cell.

[220] Caldwell R.B., McLaughlin B.J. (1985). Freeze-fracture study of filipin binding in photoreceptor outer segments and pigment epithelium of dystrophic and normal retinas. J. Comp. Neurol..

[221] Ablonczy Z., Higbee D., Grey A.C., Koutalos Y., Schey K.L., Crouch R.K. (2013). Similar molecules spatially correlate with lipofuscin and N-retinylidene-N-retinylethanolamine in the mouse but not in the human retinal pigment epithelium. Arch. Biochem. Biophys..

[222] Bhosale P., Serban B., Bernstein P.S. (2009). Retinal carotenoids can attenuate formation of A2E in the retinal pigment epithelium. Arch. Biochem. Biophys..

[223] Zemski Berry K.A., Gordon W.C., Murphy R.C., Bazan N.G. (2014). Spatial organization of lipids in the human retina and optic nerve by MALDI imaging mass spectrometry. J. Lipid Res..

[224] Pallitto P., Ablonczy Z., Jones E.E., Drake R.R., Koutalos Y., Crouch R.K. (2015). A2E and lipofuscin distributions in macaque retinal pigment epithelium are similar to human. Photochem. Photobiol. Sci..

[225] Starnes A.C., Huisingh C., McGwin G., Sloan K.R., Ablonczy Z., Smith R.T. (2016). Multi-nucleate retinal pigment epithelium cells of the human macula exhibit a characteristic and highly specific distribution. Vis. Neurosci..

[226] Bermond K., Wobbe C., Tarau I.S., Heintzmann R., Hillenkamp J., Curcio C.A. (2020). Autofluorescent granules of the human retinal pigment epithelium: phenotypes, intracellular distribution, and age-related topography. Invest. Ophthalmol. Vis. Sci..

[227] De La Paz M.A., Anderson R.E. (1992). Lipid peroxidation in rod outer segments. Role of hydroxyl radical and lipid hydroperoxides. Invest. Ophthalmol. Vis. Sci..

[228] Ohishi K., Zhang X.M., Moriwaki S., Hiramitsu T., Matsugo S. (2006). In the presence of ferritin, visible light induces lipid peroxidation of the porcine photoreceptor outer segment. Free Radic. Res..

[229] Organisciak D.T., Vaughan D.K. (2010). Retinal light damage: mechanisms and protection. Prog. Retin. Eye Res..

[230] Song D., Song Y., Hadziahmetovic M., Zhong Y., Dunaief J.L. (2012). Systemic administration of the iron chelator deferiprone protects against light-induced photoreceptor degeneration in the mouse retina. Free Radic. Biol. Med..

[231] Fu J., Lam T.T., Tso M.O. (1992). Dexamethasone ameliorates retinal photic injury in albino rats. Exp. Eye Res..

[232] Li Z.L., Lam S., Tso M.O. (1991). Desferrioxamine ameliorates retinal photic injury in albino rats. Curr. Eye Res..

[233] Hadziahmetovic M., Pajic M., Grieco S., Song Y., Song D., Li Y. (2012). The oral iron chelator deferiprone protects against retinal degeneration induced through diverse mechanisms. Transl. Vis. Sci. Technol..

[234] Song D., Dunaief J.L. (2013). Retinal iron homeostasis in health and disease. Front. Aging Neurosci..

[235] Tanito M., Elliott M.H., Kotake Y., Anderson R.E. (2005). Protein modifications by 4-hydroxynonenal and 4-hydroxyhexenal in light-exposed rat retina. Invest. Ophthalmol. Vis. Sci..

[236] Salomon R.G., Bi W. (2015). Isolevuglandin adducts in disease. Antioxid. Redox Signal.

[237] Zhang M., Li W., Li T. (2011). Generation and detection of levuglandins and isolevuglandins in vitro and in vivo. Molecules.

[238] Natoli R., Jiao H., Barnett N.L., Fernando N., Valter K., Provis J.M. (2016). A model of progressive photo-oxidative degeneration and inflammation in the pigmented C57BL/6J mouse retina. Exp. Eye Res..

[239] Kapphahn R.J., Richards M.J., Ferrington D.A., Fliesler S.J. (2019). Lipid-derived and other oxidative modifications of retinal proteins in a rat model of Smith-Lemli-Opitz syndrome. Exp. Eye Res..

[240] Charvet C.D., Saadane A., Wang M., Salomon R.G., Brunengraber H., Turko I.V. (2013). Pretreatment with pyridoxamine mitigates isolevuglandin-associated retinal effects in mice exposed to bright light. J. Biol. Chem..

[241] Cronin T., Lyubarsky A., Bennett J. (2012). Dark-rearing the rd10 mouse: implications for therapy. Adv. Exp. Med. Biol..

[242] Chen J., Simon M.I., Matthes M.T., Yasumura D., LaVail M.M. (1999). Increased susceptibility to light damage in an arrestin knockout mouse model of Oguchi disease (stationary night blindness). Invest. Ophthalmol. Vis. Sci..

[243] Collin G.B., Gogna N., Chang B., Damkham N., Pinkney J., Hyde L.F. (2020). Mouse models of inherited retinal degeneration with photoreceptor cell loss. Cells.

[244] Sui G.Y., Liu G.C., Liu G.Y., Gao Y.Y., Deng Y., Wang W.Y. (2013). Is sunlight exposure a risk factor for age-related macular degeneration? A systematic review and meta-analysis. Br. J. Ophthalmol..

[245] Delcourt C., Cougnard-Gregoire A., Boniol M., Carriere I., Dore J.F., Delyfer M.N. (2014). Lifetime exposure to ambient ultraviolet radiation and the risk for cataract extraction and age-related macular degeneration: the Alienor Study. Invest. Ophthalmol. Vis. Sci..

[246] Kooijman A.C. (1983). Light distribution on the retina of a wide-angle theoretical eye. J. Opt. Soc. Am..

[247] Pflibsen K.P., Pomerantzeff O., Ross R.N. (1988). Retinal illuminance using a wide-angle model of the eye. J. Opt. Soc. Am. A.

[248] Poli G., Biasi F., Leonarduzzi G. (2013). Oxysterols in the pathogenesis of major chronic diseases. Redox Biol..

[249] Rodriguez I.R., Fliesler S.J. (2009). Photodamage generates 7-keto- and 7-hydroxycholesterol in the rat retina via a free radical-mediated mechanism. Photochem. Photobiol..

[250] Hahn P., Dentchev T., Qian Y., Rouault T., Harris Z.L., Dunaief J.L. (2004). Immunolocalization and regulation of iron handling proteins ferritin and ferroportin in the retina. Mol. Vis..

[251] Chaudhary K., Promsote W., Ananth S., Veeranan-Karmegam R., Tawfik A., Arjunan P. (2018). Iron overload accelerates the progression of diabetic retinopathy in association with increased retinal renin expression. Sci. Rep..

[252] Mendes-Jorge L., Ramos D., Valenca A., Lopez-Luppo M., Pires V.M.R., Catita J. (2017). Correction: L-Ferritin binding to Scara5: a new iron traffic pathway potentially implicated in retinopathy. PLoS One.

[253] Omarova S., Charvet C.D., Reem R.E., Mast N., Zheng W., Huang S. (2012). Abnormal vascularization in mouse retina with dysregulated retinal cholesterol homeostasis. J. Clin. Invest..

[254] Neekhra A., Luthra S., Chwa M., Seigel G., Gramajo A.L., Kuppermann B.D. (2007). Caspase-8, -12, and -3 activation by 7-ketocholesterol in retinal neurosensory cells. Invest. Ophthalmol. Vis. Sci..

[255] Luthra S., Fardin B., Dong J., Hertzog D., Kamjoo S., Gebremariam S. (2006). Activation of caspase-8 and caspase-12 pathways by 7-ketocholesterol in human retinal pigment epithelial cells. Invest. Ophthalmol. Vis. Sci..

[256] Pfeffer B.A., Xu L., Porter N.A., Rao S.R., Fliesler S.J. (2016). Differential cytotoxic effects of 7-dehydrocholesterol-derived oxysterols on cultured retina-derived cells: dependence on sterol structure, cell type, and density. Exp. Eye Res..

[257] Wu M., Yang S., Elliott M.H., Fu D., Wilson K., Zhang J. (2012). Oxidative and endoplasmic reticulum stresses mediate apoptosis induced by modified LDL in human retinal Muller cells. Invest. Ophthalmol. Vis. Sci..

[258] Indaram M., Ma W., Zhao L., Fariss R.N., Rodriguez I.R., Wong W.T. (2015). 7-Ketocholesterol increases retinal microglial migration, activation, and angiogenicity: a potential pathogenic mechanism underlying age-related macular degeneration. Sci. Rep..

[259] Fu D., Yu J.Y., Wu M., Du M., Chen Y., Abdelsamie S.A. (2014). Immune complex formation in human diabetic retina enhances toxicity of oxidized LDL towards retinal capillary pericytes. J. Lipid Res..

[260] Honjo M., Nakamura K., Yamashiro K., Kiryu J., Tanihara H., McEvoy L.M. (2003). Lectin-like oxidized LDL receptor-1 is a cell-adhesion molecule involved in endotoxin-induced inflammation. Proc. Natl. Acad. Sci. U.S.A..

[261] Baba T., Bhutto I.A., Merges C., Grebe R., Emmert D., McLeod D.S. (2010). A rat model for choroidal neovascularization using subretinal lipid hydroperoxide injection. Am. J. Pathol..

[262] Xu L., Sheflin L.G., Porter N.A., Fliesler S.J. (2012). 7-Dehydrocholesterol-derived oxysterols and retinal degeneration in a rat model of Smith-Lemli-Opitz syndrome. Biochim. Biophys. Acta.

[263] Yang C., Xie L., Gu Q., Qiu Q., Wu X., Yin L. (2019). 7-Ketocholesterol disturbs RPE cells phagocytosis of the outer segment of photoreceptor and induces inflammation through ERK signaling pathway. Exp. Eye Res..

[264] Heo G.Y., Bederman I., Mast N., Liao W.L., Turko I.V., Pikuleva I.A. (2011). Conversion of 7-ketocholesterol to oxysterol metabolites by recombinant CYP27A1 and retinal pigment epithelial cells. J. Lipid Res..

[265] Nakano A., Kawashima H., Miyake Y., Zeniya T., Yamamoto A., Koshino K. (2018). (123)I-Labeled oxLDL is widely distributed throughout the whole body in mice. Nucl. Med. Mol. Imaging.

[266] Kato R., Mori C., Kitazato K., Arata S., Obama T., Mori M. (2009). Transient increase in plasma oxidized LDL during the progression of atherosclerosis in apolipoprotein E knockout mice. Arterioscler. Thromb. Vasc. Biol..

[267] Que X., Hung M.Y., Yeang C., Gonen A., Prohaska T.A., Sun X. (2018). Oxidized phospholipids are proinflammatory and proatherogenic in hypercholesterolaemic mice. Nature.

[268] Fourgeux C., Martine L., Pasquis B., Maire M.A., Acar N., Creuzot-Garcher C. (2012). Steady-state levels of retinal 24S-hydroxycholesterol are maintained by glial cells intervention after elevation of intraocular pressure in the rat. Acta Ophthalmol..

[269] Xu L., Liu W., Sheflin L.G., Fliesler S.J., Porter N.A. (2011). Novel oxysterols observed in tissues and fluids of AY9944-treated rats: a model for Smith-Lemli-Opitz syndrome. J. Lipid Res..

[270] Xu L., Korade Z., Rosado D.A., Mirnics K., Porter N.A. (2013). Metabolism of oxysterols derived from nonenzymatic oxidation of 7-dehydrocholesterol in cells. J. Lipid Res..

[271] Vaughan D.K., Peachey N.S., Richards M.J., Buchan B., Fliesler S.J. (2006). Light-induced exacerbation of retinal degeneration in a rat model of Smith-Lemli-Opitz syndrome. Exp. Eye Res..

[272] Organisciak D.T., Darrow R.M., Jiang Y.I., Marak G.E., Blanks J.C. (1992). Protection by dimethylthiourea against retinal light damage in rats. Invest. Ophthalmol. Vis. Sci..

[273] Organisciak D.T., Darrow R.A., Barsalou L., Darrow R.M., Lininger L.A. (1999). Light-induced damage in the retina: differential effects of dimethylthiourea on photoreceptor survival, apoptosis and DNA oxidation. Photochem. Photobiol..

[274] Richards M.J., Nagel B.A., Fliesler S.J. (2006). Lipid hydroperoxide formation in the retina: correlation with retinal degeneration and light damage in a rat model of Smith-Lemli-Opitz syndrome. Exp. Eye Res..

[275] Fliesler S.J., Peachey N.S., Herron J., Hines K.M., Weinstock N.I., Ramachandra Rao S. (2018). Prevention of retinal degeneration in a rat model of smith-lemli-opitz syndrome. Sci. Rep..

[276] Fliesler S.J. (2013). Antioxidants: the missing key to improved therapeutic intervention in Smith-Lemli-Opitz syndrome?. Hereditary Genet..

[277] Andersson H.C., Kratz L., Kelley R. (2002). Desmosterolosis presenting with multiple congenital anomalies and profound developmental delay. Am. J. Med. Genet..

[278] Zerenturk E.J., Sharpe L.J., Ikonen E., Brown A.J. (2013). Desmosterol and DHCR24: unexpected new directions for a terminal step in cholesterol synthesis. Prog. Lipid Res..

[279] Cenedella R.J. (1983). Source of cholesterol for the ocular lens, studied with U18666A: a cataract-producing inhibitor of lipid metabolism. Exp. Eye Res..

[280] Cenedella R.J. (2009). Cholesterol synthesis inhibitor U18666A and the role of sterol metabolism and trafficking in numerous pathophysiological processes. Lipids.

[281] Fliesler S.J., Richards M.J., Miller C., Peachey N.S., Cenedella R.J. (2000). Retinal structure and function in an animal model that replicates the biochemical hallmarks of desmosterolosis. Neurochem. Res..

[282] Cenedella R.J., Jacob R., Borchman D., Tang D., Neely A.R., Samadi A. (2004). Direct perturbation of lens membrane structure may contribute to cataracts caused by U18666A, an oxidosqualene cyclase inhibitor. J. Lipid Res..

[283] Sexton R.C., Panini S.R., Azran F., Rudney H. (1983). Effects of 3 beta-[2-(diethylamino)ethoxy]androst-5-en-17-one on the synthesis of cholesterol and ubiquinone in rat intestinal epithelial cell cultures. Biochemistry.

[284] Fliesler S.J., Richards M.J., Miller C.Y., Cenedella R.J. (2000). Cholesterol synthesis in the vertebrate retina: effects of U18666A on rat retinal structure, photoreceptor membrane assembly, and sterol metabolism and composition. Lipids.

[285] Lu F., Liang Q., Abi-Mosleh L., Das A., De Brabander J.K., Goldstein J.L. (2015). Identification of NPC1 as the target of U18666A, an inhibitor of lysosomal cholesterol export and Ebola infection. Elife.

[286] Quan X., Chen X., Sun D., Xu B., Zhao L., Shi X. (2016). The mechanism of the effect of U18666a on blocking the activity of 3beta-hydroxysterol Delta-24-reductase (DHCR24): molecular dynamics simulation study and free energy analysis. J. Mol. Model.

[287] Tillack T.W., Kinsky S.C. (1973). A freeze-etch study of the effects of filipin on liposomes and human erythrocyte membranes. Biochim. Biophys. Acta.

[288] Andrews L.D., Cohen A.I. (1979). Freeze-fracture evidence for the presence of cholesterol in particle-free patches of basal disks and the plasma membrane of retinal rod outer segments of mice and frogs. J. Cell Biol..

[289] Andrews L.D., Cohen A.I. (1981). Freeze-fracture studies of the structure of rod outer segment membranes: new observations regarding the distribution of particle-free patches and the location of the fracture planes in conventionally prepared retinas. Exp. Eye Res..

[290] Nishikawa M., Nojima S., Akiyama T., Sankawa U., Inoue K. (1984). Interaction of digitonin and its analogs with membrane cholesterol. J. Biochem..

[291] Magalhaes M.M., Coimbra A. (1972). The rabbit retina Muller cell. A fine structural and cytochemical study. J. Ultrastruct. Res..

[292] Maxfield F.R., Wustner D. (2012). Analysis of cholesterol trafficking with fluorescent probes. Methods Cell Biol.

[293] Maekawa M., Yang Y., Fairn G.D. (2016). Perfringolysin O Theta Toxin as a Tool to Monitor the Distribution and Inhomogeneity of Cholesterol in Cellular Membranes. Toxins (Basel)..

[294] Mobius W., Ohno-Iwashita Y., van Donselaar E.G., Oorschot V.M., Shimada Y., Fujimoto T. (2002). Immunoelectron microscopic localization of cholesterol using biotinylated and non-cytolytic perfringolysin O. J. Histochem. Cytochem..

[295] Maekawa M., Fairn G.D. (2015). Complementary probes reveal that phosphatidylserine is required for the proper transbilayer distribution of cholesterol. J. Cell Sci..

[296] Tan L., Cho K.J., Kattan W.E., Garrido C.M., Zhou Y., Neupane P. (2019). Acylpeptide hydrolase is a novel regulator of KRAS plasma membrane localization and function. J. Cell Sci..

[297] Maekawa M., Lee M., Wei K., Ridgway N.D., Fairn G.D. (2016). Staurosporines decrease ORMDL proteins and enhance sphingomyelin synthesis resulting in depletion of plasmalemmal phosphatidylserine. Sci. Rep..

[298] Jao C.Y., Nedelcu D., Lopez L.V., Samarakoon T.N., Welti R., Salic A. (2015). Bioorthogonal probes for imaging sterols in cells. Chembiochem.

[299] Peyrot S.M., Nachtergaele S., Luchetti G., Mydock-McGrane L.K., Fujiwara H., Scherrer D. (2014). Tracking the subcellular fate of 20(s)-hydroxycholesterol with click chemistry reveals a transport pathway to the Golgi. J. Biol. Chem..

